# Advancing Analytical Technologies for Rapid Food Freshness Detection: A Comprehensive Review

**DOI:** 10.3390/foods15183263

**Published:** 2026-09-15

**Authors:** Lin Cheng, Yangyang Lu, Yi Shao, Wei Song

**Affiliations:** 1 Modern Industrial College of Traditional Chinese Medicine and Health, Lishui University, 1 Xueyuan Road, Lishui 323000, China; 2 Institute for Agro-food Standards and Testing Technology, Shanghai Academy of Agricultural Sciences, 1000 Jinqi Road, Shanghai 201403, China

**Keywords:** food freshness, rapid detection, sensory simulation, biosensor, spectral imaging

## Abstract

Food freshness is critical to ensuring food safety, minimizing food waste, and improving industrial efficiency. Traditional sensory evaluation and laboratory-based detection methods suffer from inherent limitations, including strong subjectivity, time-consuming procedures, and destructive sampling, which make it difficult to meet the requirements of modern supply chains for rapid, non-destructive, and real-time monitoring. This review comprehensively summarizes the mainstream research directions and recent advances in food freshness monitoring technologies across three complementary directions: (1) biomimetic or instrumental systems based on digital identification of food odors, tastes, and appearance characteristics, including electronic noses, electronic tongues, and machine vision technology; (2) biosensor-based technologies for ultrasensitive recognition of specific biomolecules (metabolites, proteins, and nucleic acids); (3) spectroscopic and imaging techniques for “fingerprint” capture and quantitative analysis of internal components. In addition, the preparation principles, instrumentation, and applications are stated in each section, along with critical performance aspects such as stability and anti-interference capability in complex matrices. We hope this review can provide a comprehensive understanding of sustainable and real-time food quality control for the advancement of food freshness assessment.

## 1. Introduction

Food freshness serves as a fundamental indicator of edible quality and economic value, closely linked to consumer acceptance, resource utilization, and industrial sustainability [1]. Spoilage causes staggering food loss and waste, incurring enormous economic and environmental costs [2], while pathogenic microorganisms and hazardous metabolites such as biogenic amines and sulfides pose potential health risks [3]. Freshness indicators reflect freshness deterioration, underscoring the need for accurate, rapid, and field-deployable freshness assessment systems, such as total volatile basic nitrogen (TVB-N), total viable count (TVC), microorganisms, and biogenic amines. As core freshness indicators, TVB-N reflects protein degradation driven by endogenous enzymes and spoilage microbes; TVC quantifies overall proliferation of spoilage-associated microorganisms; biogenic amines are metabolic by-products generated during food spoilage. Meanwhile, shelf-life describes the time window maintaining acceptable organoleptic and physicochemical quality under defined storage conditions. Notably, these indicators describe freshness deterioration and spoilage trends, and do not inherently equate to pathogenic food-safety hazards.

Traditional freshness assessment requires advanced instruments and skilled operators, as well as tedious sample preparation processes, such as sensory, physicochemical, and microbiological detection techniques [4,5]. While the importance of the above methods is undeniable, analytical and destructive techniques are time-consuming and expensive, adversely impact the environment, and may require advancced instrumentation. Hence, rapid, reliable, less expensive, and less polluting methods for freshness assessment are required to meet the modern food industry’s urgent demand for online, real-time, non-destructive monitoring, especially for dynamic quality monitoring across the entire chain from production to retail. This has prompted researchers to turn to developing rapid detection methods based on advanced sensing and information processing technologies, and related research has experienced explosive growth in the past decade [6,7].

To date, rapid food freshness detection technologies have formed a trend of mutual intersection, jointly propelling the field towards intelligence and precision. Examples include sensory simulation technologies such as electronic nose, electronic tongue, and machine vision [8,9,10]; biosensing technologies based on enzymes, antibodies, nucleic acid aptamers, etc. [11,12,13,14,15]; and spectroscopy and imaging technologies such as infrared spectroscopy, Hyperspectral Imaging, and Raman spectroscopy, combined with optical, electrochemical, machine learning, and other technologies to achieve rapid analysis and detection [16,17,18,19]. In recent years, the introduction of artificial intelligence (especially deep learning) and multi-source information fusion technologies has greatly enhanced the ability of these technologies to extract features from complex data and build predictive models [20,21].

Several reviews have comprehensively investigated analytical technologies for food freshness monitoring, including electronic noses/tongues, optical sensors, Hyperspectral Imaging, spectral methods, and their integration with deep learning and artificial intelligence, but few have provided a comprehensive cross-platform comparison that critically evaluates their relative performance and translational potential. This review aims to comprehensively organize the latest advances and development trends in the field of rapid detection of food freshness. This article elaborates on sensory simulation technologies, including the electronic nose, electronic tongue, and machine vision. It then delves into biosensing strategies employing diverse biological recognition elements such as enzymes, antibodies, nucleic acids, and microorganisms. Subsequently, advanced detection approaches based on spectroscopy and imaging, as well as their integration with artificial intelligence, are comprehensively explored. Finally, this review elaborates on the intrinsic performance-versus-practice trade-offs for each technology, which will provide an in-depth analysis of the main current challenges and offer an outlook on future research directions, especially the development of intelligent perception systems driven by the deep integration of advanced functional materials and artificial intelligence.

## 2. Rapid Detection Technologies Based on Sensory Simulation

### 2.1. Electronic Nose Technology

Food spoilage during storage releases characteristic volatile organic compounds, providing a theoretical basis for odor analysis [22,23]. The electronic nose captures these odor molecules through a gas sensor array, converts them into electrical signals, and combines them with pattern recognition algorithms to achieve rapid, non-destructive discrimination of freshness for meat and other foods [24]. Electronic nose technology in the field of food quality detection is undergoing significant technological evolution, with analysis methods gradually shifting from traditional machine learning to more complex deep-learning architectures. Meanwhile, detection targets have developed from static quality classification to dynamic time-series prediction of freshness and shelf-life assessment.

**Figure 1:**
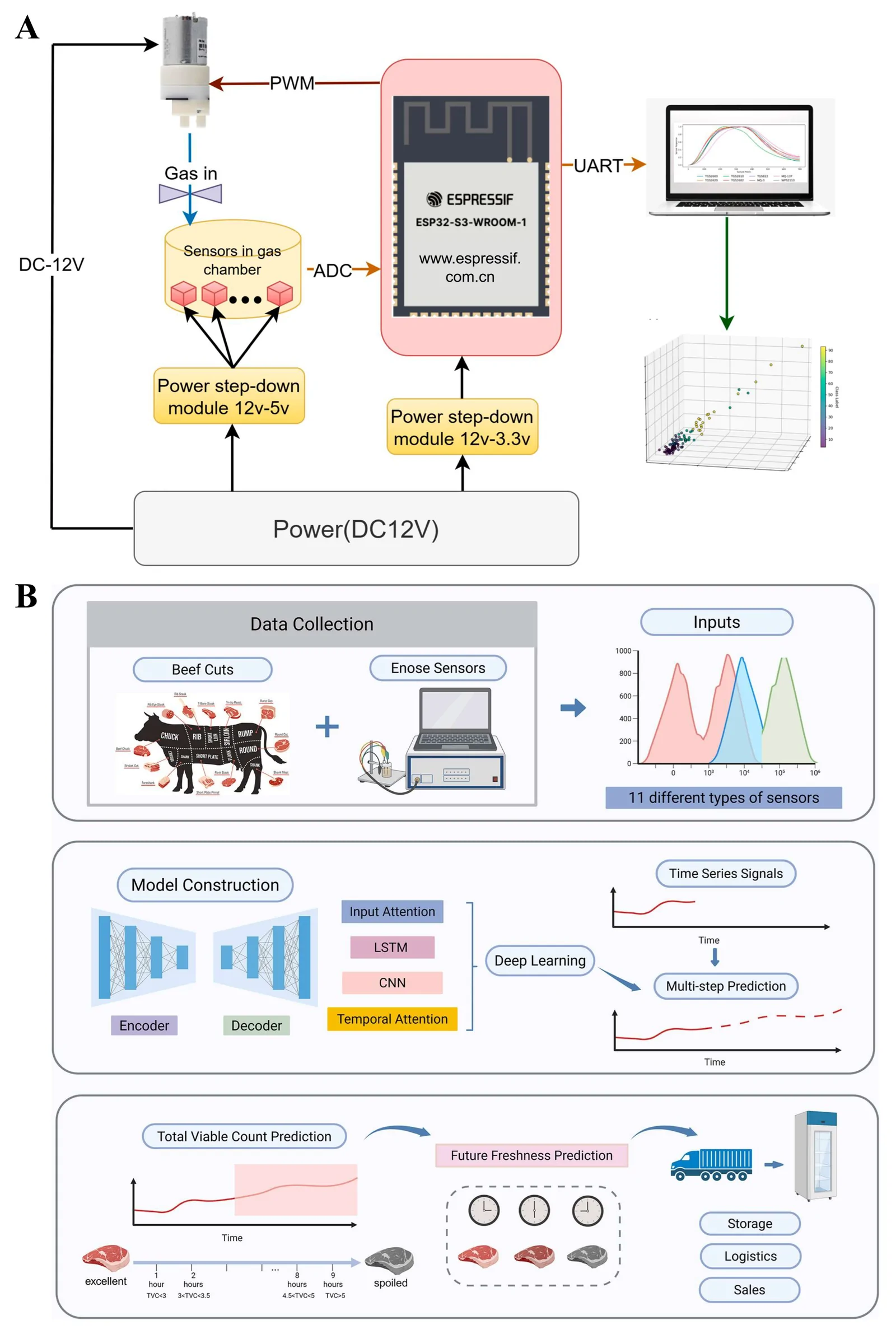
(**A**) Overall framework of an electronic nose freshness detection system based on traditional machine learning. Adapted with permission from Ref. [26]. Copyright 2026, Springer Nature. (**B**) Overall workflow for predicting future freshness of meat products by combining an electronic nose with time series analysis. Adapted with permission from Ref. [30]. Copyright 2025, Elsevier.

Traditional machine learning-based methods focus on combining electronic nose data with chemical analysis techniques and classical machine learning algorithms for pattern recognition, offering strong interpretability and technical maturity. For example, Wang et al. constructed an efficient non-destructive freshness-evaluation model for oysters using a self-built electronic nose combined with a support vector machine and Random Forest algorithms; a gas chromatography-mass spectrometer (GC-MS) was employed in parallel to characterize volatile spoilage markers and interpret sensor response mechanisms [25] (Table 1). Zhou et al. [26] designed a custom-built electronic-nose system for apple spoilage assessment (as shown in Figure 1A). Coupled with the proposed three-phase feature integration (TPFI) feature-engineering approach and machine-learning classifiers, their TPFI-PCA-MLP model achieved 99.6% accuracy in discriminating apples across six storage time-points, demonstrating the promising capability of machine-learning-based electronic noses for fruit-storage monitoring [26].

**Table 1:** Summary of sensory simulation technologies for food freshness monitoring.

Sensor Technology	Food Matrix	Analytes	Response Time	Accuracy	R^2^/Precision	Repeatability/Reproducibility/Robustness	Chemometric Methods	LOD/LOQ	External Validation	Destructive	Ref.
Electronic Nose Technology	Oyster	Freshness classification; VOCs distribution (alcohols, acids, aldehydes, heterocyclic compounds)	/	SVM/RF > 90%	/	/	PCA, LDA, SVM, RF, PCA	/	No	No	[25]
	Rice	Quality identification under different storage temperatures (25 °C, 40 °C) and periods	60 s detection time; 30 min headspace accumulation	92.58–97.42%	F1-score: 92.74–97.64%.	/	MSAM-Net; CNN-Net, SE-Net, ECA-Net, CA-Net, CBAM-Net, SK-Net	/	No	No	[27]
	Pork	TVB-N	0.159 s	/	R^2^*p* = 0.9373, RMSEP = 0.4897 mg/100 g, RPD = 3.5454	/	HFA-Net fusion model	/	Yes	No	[28]
	Mutton	TVB-N	/	/	R = 0.920; RPD = 3.59	/	IMCNN	/	Yes	No	[29]
	Beef	TVC	/	/	R^2^ = 0.95, RMSE = 0.097 (1 h); R^2^ > 0.859 (9 h)	Validated on 12 beef regions for robust generalization across heterogeneous tissue-specific spoilage patterns	DARNN, LSTM, TCN, Transformer_LSTM, ANN, RNN, MainModel	/	Yes	No	[30]
	20 types of foods	Hydrogen sulfide, ammonia, ethanol, ethylene, ethanol, hydrogen		97.3	Macro-f1: 0.972	/	CNN	0.05 ppm–10 ppm	No	Yes	[31]
	Chicken breast	Odor data	/	ResNet: 99.70%; SVM: 94.33%	/	/	Transfer learning (AlexNet, GoogLeNet, ResNet); time-series-to-image conversion (scatter plot, fitting curve, feature heat map); KNN, RF, SVM for comparison	/	No	No	[32]
	Oyster	TVC, TVB-N, BAs	/	81.2%	/	/	PriorBoost-CNN-LSTM	/	Yes	No	[33]
Electronic Tongue Technology	Crucian carp	TVC	/	/	R_pre_ = 0.993, RMSEP = 0.211 cfu/g	/	PLS-R, SVR, BP-NN	/	/	Yes	[34]
	Crucian carp	TVCs	/	/	BP-NN: R_pre_ = 0.993, RMSEP = 0.211 ln CFU/g (superior to PLS and SVR)	/	PLS regression, SVR, BP-NN	/	Yes	Yes	[35]
	Pork loin	Monitoring physicochemical and microbiological changes: pH, microbial count, ATP degradation compounds (IMP, Ino, Hx)	≥5 min for electrode equilibrium	/	PCA: PC1 + PC2 = 86.57% variance	/	PCA, MLP ANN, Fuzzy Artmap	/	Yes	Yes	[36]
	Pork	Volatile organic compounds like alcohols, aldehydes, and hydrocarbons	/	/	/	/	Multivariate statistical analysis	/	Yes	No	[37]
	Satsuma mandarin, sweet orange and mixed citrus juices	Volatile components, sensory attributes	/	100% (LDA)	Training set: R^2^ > 0.994 (sensory), R^2^ > 0.992) (volatiles); test set: R^2^ > 0.983) (sensory), R^2^ > 0.990) (volatiles)	/	LDA, PLS-R, Random Forest	/	/	No	[38]
	Pork	Methane, alcohols, ketones, and aldehydes; taste for richness, bitterness, astringency, aftertaste, astringency, and sourness	/	/	/	/	Multivariate analysis	/	Yes	No	[39]
Machine Vision Technology	Tomato	Improved frequency-tuned (FT) visual saliency detection	0.0326 s	98.38%	Precision = 98.69%; recall = 98.32%	/	ResNet34 model	/	/	No	[40]
	Pork and chicken	TVB-N	/	/	pork: R^2^ = 0.91, RMSEP = 1.21; chicken: R^2^ = 0.94, RMSEP = 0.98	/	PCA, SVM, PLS	/	Yes	Yes	[41]
	Meat (pork/chicken)	pH	/	95.3%	/	/	CNN	/	/	Yes	[42]
	Shrimp and fish	VOCs	/	99%	/	Stable and consistent colorimetric change even at high RH (60%)	CNN	LOD: ammonia = 2 ppm, dimethylamine = 21 ppm, TMA = 3 ppm, cadaverine = 1 ppm, putrescine = 1 ppm	/	/	[43]
	Meat/fish	Gelatin methacryloyl	30 s	96.2%	/	/	CNN with VGG16 architecture; marker-based watershed algorithm	/	/	No	[44]

Deep-learning-model-based methods primarily construct end-to-end deep neural networks to directly process the raw time-series signals or transformed data collected by the electronic nose, automatically extracting deep features and performing classification or prediction. Its advantage lies in the ability to automatically learn complex non-linear relationships, particularly excelling at identifying key patterns from high-dimensional data. Built around a Multi-branch Self-attention Module-Net (MSAM-Net) for odor-feature extraction, this model yielded over 96% classification accuracy when identifying rice quality under varied storage conditions [27]. The Hybrid Fusion Attention Network (HFA-Net) realized deep multi-modal fusion of electronic-nose and fluorescence Hyperspectral Imaging data for predicting pork TVB-N, yielding high predictive performance with R^2^*p* = 0.9373 (coefficient of determination for the prediction set) [28]. Comparably, the input-modified convolutional neural network (IMCNN) achieved reliable TVB-N prediction for mutton R^2^*p* = 0.920), further illustrating the merits of multi-modal data fusion approaches [29].

To further reveal the correlation between sensing mechanisms and spoilage processes, sequential prediction and mechanism analysis are developed to predict subsequent quality states and go beyond real-time diagnosis. As shown in Figure 1B, a multi-step time-series forecasting framework was constructed by Li et al. [30]. This approach realizes robust long-term prediction of beef quality changes up to 9 h ahead, maintaining R^2^ (determination coefficient) > 0.859 across twelve beef tissue regions. Ren et al. developed a MEMS-MOX electronic nose system, which constructs abstract odor maps by integrating steady-state and transient sensor response information [31]. The multi-category food freshness classification accuracy reaches 97.3% for 20 food types, with a 6.5% accuracy gain contributed by time-series features, verifying their critical role in boosting recognition performance. In addition, electronic-nose time-series signals are innovatively converted into image-form inputs. Pre-trained convolutional neural networks with transfer-learning strategies are adopted for classification, achieving 99.70% accuracy in evaluating chicken breast freshness [32]. Meanwhile, the PriorBoost-CNN-LSTM architecture integrates physicochemical prior knowledge into network weights for oyster freshness assessment. Against the baseline vanilla CNN-LSTM model, it improved the hourly level prediction accuracy of oyster freshness under varied storage temperatures from 69.7% to 81.2% [33]. Advanced time-series feature mining, transfer-learning deployment, and prior-knowledge-enhanced modeling have thus emerged as key research avenues to promote the reliability and practical deployment of electronic-nose-based detection systems.

### 2.2. Electronic Tongue Technology

The electronic tongue is a biomimetic taste analysis technology that simulates biological taste receptors to convert chemical information from extracts into electrical signals and uses a pattern recognition system for objective quantitative assessment. Since its development in the 1980s, this technology has evolved into a technical system consisting of sensors, signal acquisition, and pattern recognition. It is widely applied to the freshness and maturity detection of various foods, with objectivity significantly superior to traditional sensory evaluation [45].

**Figure 2:**
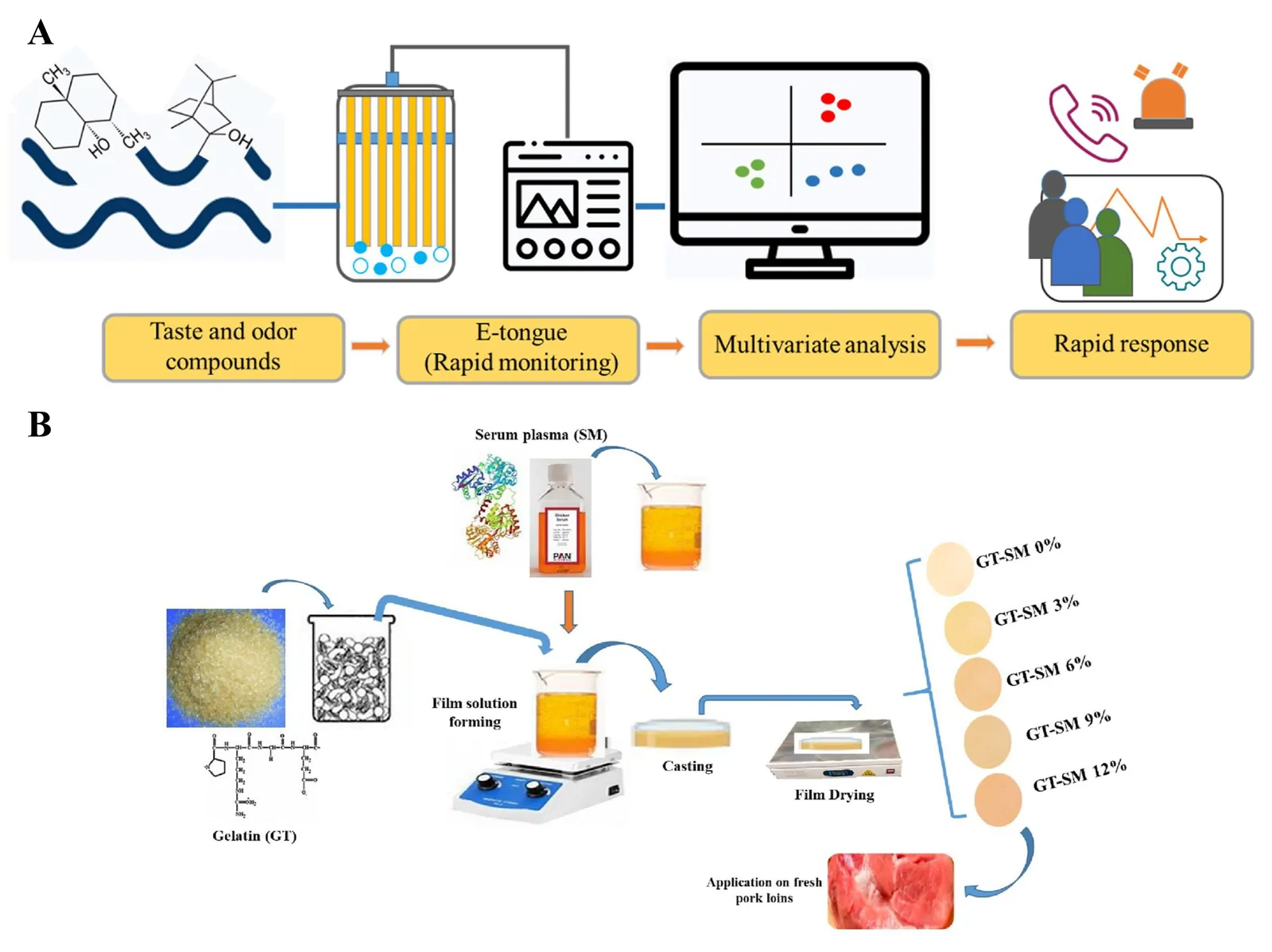
(**A**) Schematic diagram of electronic tongue workflow based on multivariate statistical recognition. Adapted with permission from Ref. [36]. Copyright 2011, John Wiley and Sons. (**B**) Schematic diagram of an integrated smart packaging system using an electronic tongue as a verification tool. Adapted with permission from Ref. [39]. Copyright 2024, Elsevier.

In fact, traditional multivariate statistical recognition-based methods primarily rely on classical chemometrics tools for dimensionality reduction, classification, or regression analysis of multidimensional data collected by the electronic tongue. For instance, support vector regression (SVR) realized the quantitative prediction of TVC, microbial freshness markers from electronic tongue data of crucian carp stored at 4 °C, while the back-propagation neural network (BP-NN) achieved superior predictive performance with an RMSEP of 0.211 cfu/g and R^2^*p* of 0.993 (Figure 2A, Table 1) [34]. This model has been applied in monitoring fish microbiology [35]. Beyond food matrices, multivariate chemometric tools such as principal component analysis and partial least squares have enabled an electronic tongue to discriminate and quantify eight taste-and-odor compounds (e.g., geosmin, 2-methylisoborneol) in natural water within 3 min at concentrations as low as 0.02 μg/L, offering a rapid alternative to the labor-intensive 1 h HS-SPME-GC-MS protocol [36]. These results indicate that electronic tongue-based approaches hold promise as alternatives to labor-intensive conventional microbial detection.

Recently, the electronic tongue is increasingly applied not as a standalone core detector but as an auxiliary tool for result verification and mechanistic analysis. For curcumin-incorporated gelatin/serum-plasma smart packaging, multi-dimensional analytical tools including electronic tongue, electronic nose and HS-SPME-GC-MS were applied to evaluate its pork-preservation performance and visual spoilage-indicating ability under different storage temperatures [37]. Likewise, the joint deployment of electronic tongue and electronic nose offers an alternative to GC-MS and human sensory evaluation for volatile-component analysis of citrus fruits and blended juices, achieving favorable classification and prediction performance [38]. As illustrated in Figure 2B, Ibeogu et al. demonstrated that edible gelatin–serum-plasma films exert positive effects on prolonging the shelf-life of fresh pork [39].

Electronic tongue technology, combined with chemometric algorithms and multi-technique fusion strategies, enables accurate and objective evaluation of food freshness and microbial quality for intelligent food detection. Nevertheless, it still faces inherent challenges including sensor drift, calibration shift, poor test reproducibility, and insufficient unified standards, which limit the model generalization and large-scale standardized application of this technology in food quality monitoring. Thus, further research is needed to enhance model generalization, reduce system costs, and promote standardized applications, so as to fully realize its potential in comprehensive food quality and safety control.

### 2.3. Machine Vision Technology

Given that color change in food freshness is an intuitive visual signal, machine vision technology captures and quantifies the external color changes objectively and efficiently [46,47]. This technology has been widely used for automatic grading, defect detection, and quality control of postharvest agricultural products. Machine vision technology has significantly improved sorting accuracy and processing efficiency by combining with machine learning methods like convolutional neural networks (CNNs).

**Figure 3:**
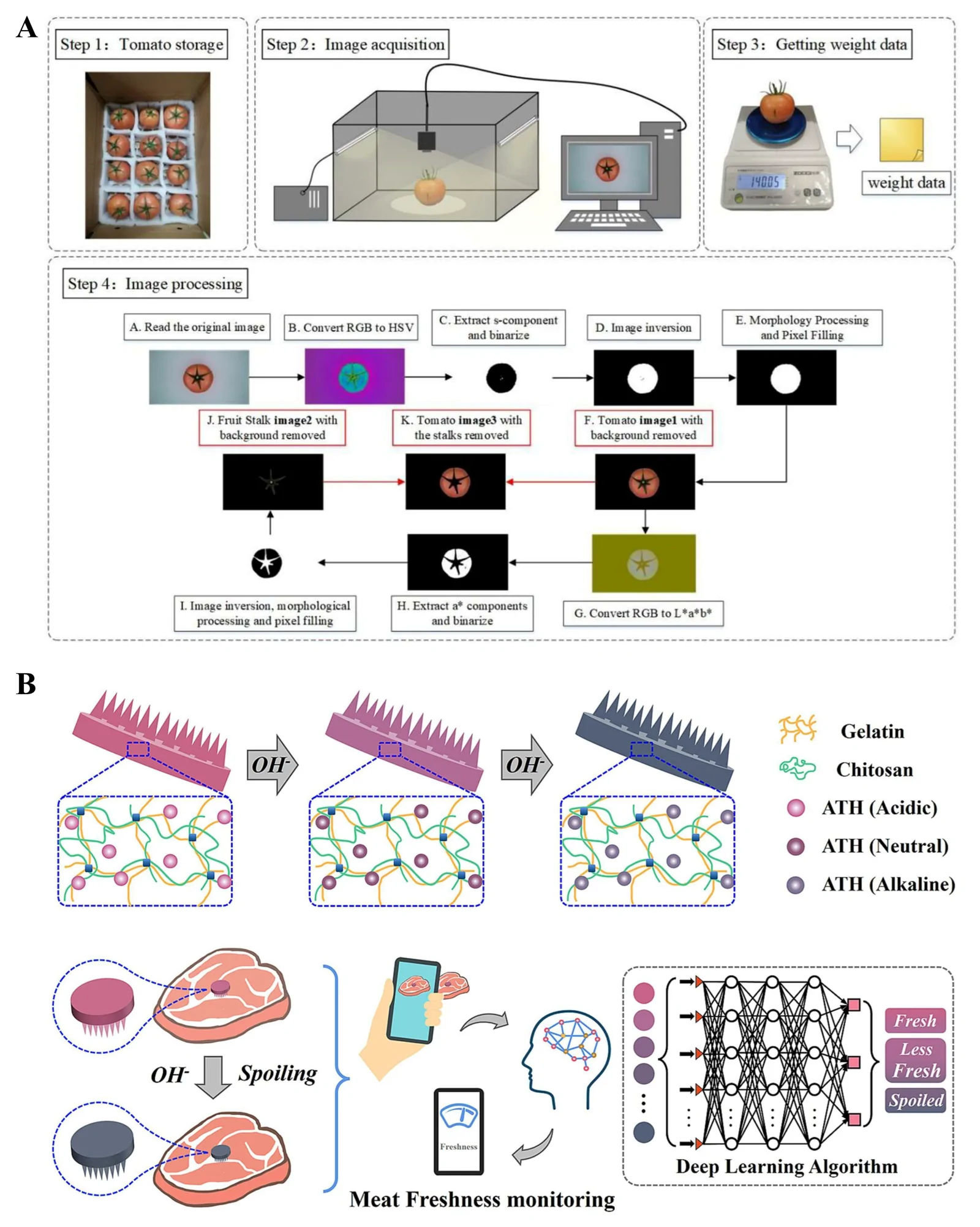
(**A**) Data acquisition and processing flow for rapid and accurate assessment of postharvest tomato freshness. Adapted with permission from Ref. [40]. Copyright 2025, John Wiley and Sons. L*a*b* represented the stytle of color converter based on CIE LAB model. (**B**) Schematic diagram of freshness detection based on a colorimetric microneedle sensor. Adapted with permission from Ref. [49]. Copyright 2025, John Wiley and Sons.

Deep-learning-based techniques enable automatic extraction of freshness-relevant features (e.g., color, texture, morphology) from food digital images. For instance, a two-stage deep-learning framework was developed to predict pork color grade with high precision by performing pork-region segmentation and accurate color-feature identification [48]. Subsequently, an improved frequency-tuned visual saliency detection method enhanced post-harvest tomato freshness recognition via visual saliency maps serving as augmented feature inputs (Figure 3A, Table 1) [40]. In more recent work, a multimodal detection system combining computer vision, electronic nose, and artificial tactile sensors was established. It delivered high-performance prediction of TVB-N for pork and chicken, yielding R^2^ of 0.91 and 0.94, respectively [41].

On the other hand, colorimetric sensor image recognition is applied in monitoring color changes in intelligent materials (e.g., smart labels, microneedles), which are sensitive to specific chemical components produced during food spoilage, such as pH, TVB-N, and volatile amines. These sensor images were indirectly interpreted as proxies for food freshness using deep-learning algorithms (Figure 3B) [49]. Among them, a smartphone-based visual detection system was developed by using pH-responsive colorimetric hydrogel microneedles, achieving in situ visual monitoring of pH variations and automatic classification of meat undergoing spoilage via CNN [42]. Meanwhile, a platform combining paper-based colorimetric sensor arrays with CNN is established to achieve the rapid and high-precision (99.25%) prediction of TVB-N value and freshness grade for shrimp and fish by recognizing odor fingerprint maps generated from spoilage [43]. Based on gelatin methacryloyl colorimetric sensing strips, Gong et al. constructed a smartphone platform for real-time and non-destructive discrimination of fish freshness grade within 30 s [44].

From the evolution of machine vision technology, traditional image processing relies on handcrafted color, texture, and morphological operators to quantify visual changes on food surfaces, offering interpretability and efficiency but limited robustness under complex illumination and individual variations. Deep learning, based on CNNs, enables end-to-end automatic feature learning and integrates multi-dimensional visual information through saliency detection and region segmentation, substantially improving grading accuracy and generalization, yet it remains confined to surface phenotypes captured by visible-light images. Multimodal approaches overcome this limitation by fusing computer vision with electronic noses, artificial tactile sensing, and colorimetric sensors (e.g., pH-responsive microneedles, paper-based arrays, and GelMA sensing strips), where vision provides spatial and morphological information, chemical sensors map spoilage markers such as pH and TVB-N, and tactile sensing supplements textural features, collectively enabling a comprehensive assessment from phenotypic observation to physicochemical essence and representing the shift from single-source to multi-source fused intelligent perception.

In summary, machine vision technology has evolved from a simple image processing tool to a comprehensive assessment system through the integration of deep learning and intelligent sensing. It enables objective grading by directly analyzing food appearances, as well as in situ indirect spoilage monitoring by translating internal chemical changes into visual signals. Nevertheless, the performance of such systems still requires further optimization to cope with complex environmental interferences. Biomimetic sensory-simulation technologies include electronic nose, electronic tongue, and machine vision, combined with chemometric and deep-learning algorithms. These technologies can realize qualitative classification of food spoilage levels and quantitative prediction of freshness indicators such as TVC and TVB-N. Compared with conventional destructive laboratory assays, these tools deliver rapid and objective outputs. Multi-sensor data fusion further offers promising potential to boost detection performance. Nevertheless, several practical constraints remain to be considered for real-world adoption.

## 3. Rapid Detection Technologies Based on Biosensing

Biosensors can be classified by two orthogonal dimensions: the signal-transduction mechanism (optical, piezoelectric, electrochemical), which converts biochemical interactions into measurable physical signals, and the biological recognition element (enzymes, antibodies, aptamers, microorganisms), which selectively binds target freshness-related analytes. Note that these two categories are not mutually exclusive. For instance, an enzyme-based biosensor can employ either optical colorimetric readout or electrochemical current output. In this review, Section 3.1, Section 3.2 and Section 3.3 are organized by signal-transduction modes, collecting sensors with varied recognition units. Section 3.4, Section 3.5, Section 3.6 and Section 3.7 are arranged according to biological recognition elements, focusing on the performance and limitations originating from the recognition moiety itself, irrespective of transducer formats.

### 3.1. Optical Biosensors

**Figure 4:**
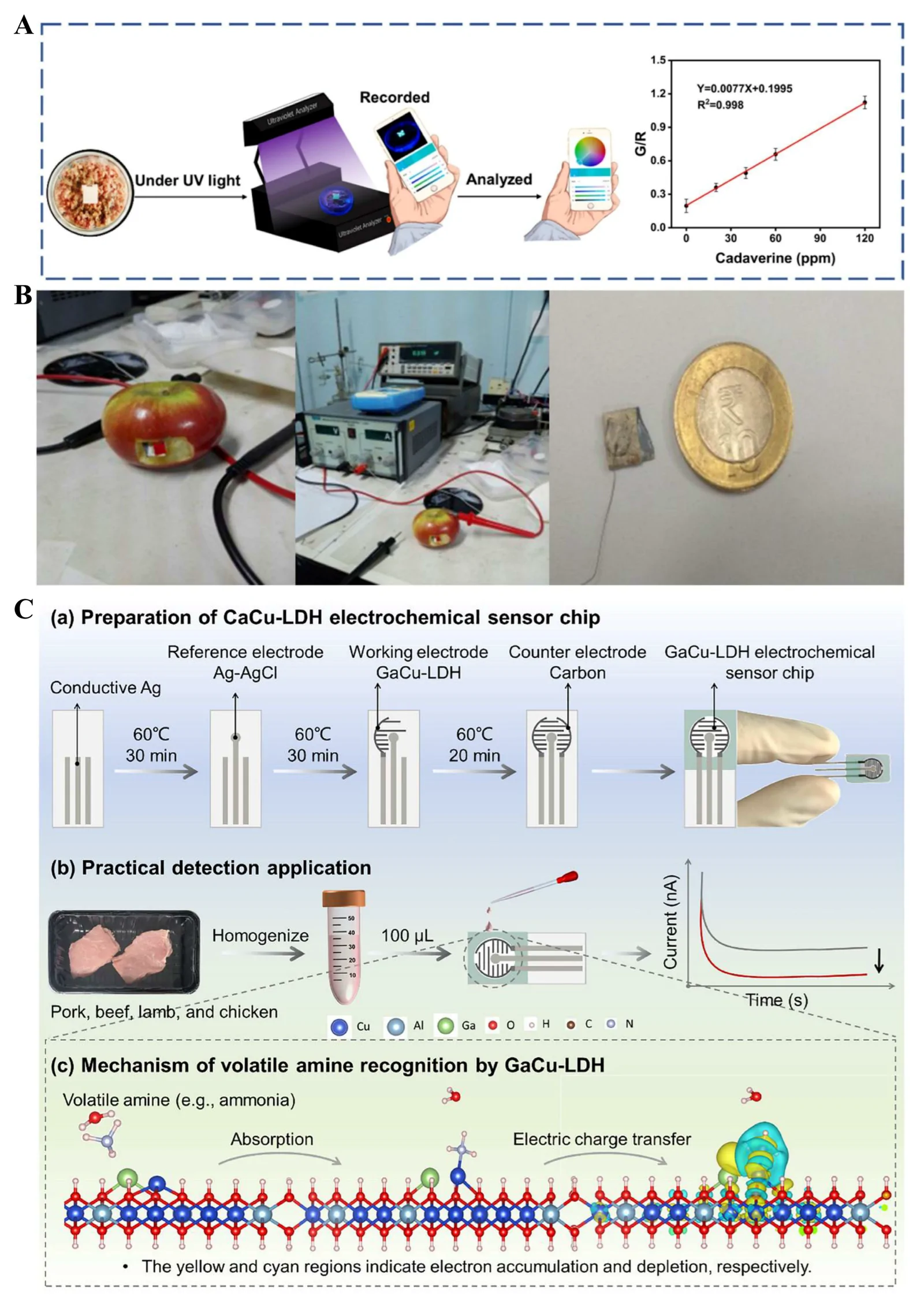
(**A**) Schematic diagram of CMDC ratiometric fluorescent sensor combined with smartphone for monitoring meat freshness. Adapted with permission from Ref. [52]. Copyright 2022, Elsevier. (**B**) Preparation and practical detection application of GaCu-LDH electrochemical biosensor chip. Adapted with permission from Ref. [62]. Copyright 2025, John Wiley and Sons.

Optical biosensors capture specific biological or chemical signals produced during food spoilage via biorecognition elements, and then convert them into measurable optical signals (e.g., color, fluorescence intensity, or refractive index), thereby enabling real-time, rapid, and non-destructive monitoring of food freshness [50,51]. For example, a ratiometric fluorescent sensor based on dicyanovinyl coumarin is designed to visually monitor the spoilage of meat by detecting cadaverine, characterized by rapid response and simplicity (Figure 4A, Table 2) [52]. Similarly, a series of naphthalene-based fluorescent molecular probes were prepared to visually monitor the freshness of pork, chicken, and shrimp, in which putrescine and cadaverine showed a rapid response (~30 s) and high sensitivity through a nucleophilic addition/elimination reaction. The detection limit (LOD) for putrescine and cadaverine was 2.69 ppm and 6.11 ppm, respectively [53]. In addition, a phenylalanine-responsive fluorescent biosensor was developed for non-invasive evaluation of fish freshness based on chemisorption of phenylalanine dehydrogenase and nicotinamide adenine dinucleotide on the graphene oxide-chitosan nanocomposite film, with a dynamic linear concentration range (0.03~0.24 mM) and an LOD of 0.02 mM by using a fluorescence spectrophotometer [51]. All of these probes were successfully integrated into a smartphone-based intelligent evaluation system for the visual monitoring of various meat products.

**Table 2:** Summary of biosensors classified by signal transduction modes for food freshness monitoring.

Sensors	Food Matrix	Analytes	Response Time	Accuracy (R^2^/Precision)	Repeatability/Reproducibility/Robustness	Chemometric Methods	LOD/LOQ	Matrix Interference	External Validation	Destructive	Ref.
Fluorescent sensor	Fish	Phenylalanine	1 min			Fluorescence spectrophotometer	LOD: 0.02 mM (linear range: 0.03–0.24 mM)	No significant interference by amines-synthesized amino acids	Excellent practicality in biological fluid samples of tuna fish (*Euthynnus affinis*)	No	[51]
Fluorescent sensor	Beef	Cadaverine	50 s	/		Ultraviolet lamps	/	/	/	No	[52]
Fluorescent sensor	Meat (pork, chicken, shrimp)	Naphthalene	30 s		Linear correlation with TVB-N	Ultraviolet-visible spectrometer and fluorescence spectrometer	LOD: putrescine, 2.69 ppm; cadaverine, 6.11 ppm	/	/	No	[53]
Colorimetric sensor	Chicken	Curcumin	Real-time	/	Reversibility in pH change	Naked-eye detection	/	/	/	No	[54]
	Chicken	pH, volatile amines	Real-time	mean color change of 34.32 ± 15.07, linear regression analysis (R^2^ = 0.797, *p* = 0.0012)	Strong antibacterial activity, water resistance, and moisture barrier	Naked-eye detection	/	/	/	No	[55]
	Pork	pH	/	/	/	/	/	/	/	No	[56]
Piezoelectric biosensors	Fruit	Ethylene	10 min	1 to 7 ppm (R = 0.9896) with RSD of <15%	RSD of <5% (*n* = 11)	/	LOD: 420 ppb	/	Correlated with gas chromatographic measurements	No	[57]
	Fish	TMA	/	/	RSD < 5% for repeated determinations of 50 ppm within 6 months	/	5–200 ppm	Completely reversible at normal temperature and relatively high humidity	Results consistent with other methods and sensory test	No	[58]
	Potatoes	Surface firmness (dry matter)	/	/	/	Arduino microcontroller board and computer	/	/	/	No	[59]
Electrochemical biosensors	Fish	XA	/	/	/	/	LOD 47.96 nM	Low interference from uric acid, bic acid, L-cysteine, glucose, and guanine	<2%RSD with spectrophotometric method	Yes	[60]
	Chilled seafood	HX and XA	/	/	Good reusability (up to 100 times)	/	0.01–10 μM	/	/	Yes	[61]
	livestock and poultry	volatile amines	4 s	/	/	/	Ammonia LOD: 5.9 μM, linear range: 0.05–0.4 mM	/	/	No	[62]
	Fish	XA, HX, MG	/	/	/	/	LOD: XA 2.9 nM (0.01–5.0 μM); HXA 5.2 nM (0.01–5.0 μM); MG 3.1 nM (0.02–2.0 μM)	/	Validated with liquid chromatography-mass spectrometry	Yes	[63]
	Fish	XA	/	10–138 μmol/L (R^2^ = 0.994); 139–330 μmol/L (R^2^ = 0.992)	/	Correlation analysis (r, *p*-value) vs. fish freshness	XA LOD: 1.036 μM	/	Correlation with XA detection in fish meat: r = 0.945, *p* < 0.01	Yes	[64]
	Fish	Volatile amine gas	1 min	/	/	/	/	/	Compared to the traditional titration method: 100 ppb vs. 18 mg/100 g and 200–300 ppb vs. 28–35 mg/100 g	No	[65]

Furthermore, an eggshell membrane coated with curcumin was designed as a suitable colorimetric sensor by effectively changing its color from yellow to red in real-time monitoring of chicken spoilage, displaying reversibility in pH change and hydrophobic nature [54]. Subsequently, a nanocomposite film sensor is further constructed based on curcumin and lemon extract, which exhibited a significantly larger color difference (ΔE = 51.85) and antibacterial, barrier, and optical properties in response to pH variations induced by chicken spoilage over 4 days [55]. In addition, an intelligent colorimetric film based on copigment-enhanced anthocyanins is designed to improve the color stability of the indicator film in detecting pork freshness through the Color Coll mobile application [56]. The above articles methodically examine the advancements in optical biosensing approaches for food freshness assessment and dissect the detailed progress from four dimensions: sensing materials, response objects, response time, and stability. These novel concepts in these studies, like green sustainability, smartphone-based portable detection systems, and antimicrobial and preservation functions, are impressive and enlightening.

Currently, optical biosensing approaches for food freshness assessment are developing from broad-spectrum responses based on natural pigments to targeted recognition enabled by chemical probes, possessing higher specificity, greater practicality, and better sustainability. Through the deep integration of sensor devices with smartphone-based image processing and data analysis, digital interpretation of detection results has been realized rapidly on-site. For industrial translation, the critical trade-off is between analytical performance and practical operability. Future development should try to bridge the gap, such as integrating fluorescent probes into biopolymer packaging films and enhancing halochromic sensors with smartphone-based colorimetric quantification, to further achieve both portability and analytical rigor.

### 3.2. Piezoelectric Biosensors

The working principle of piezoelectric biosensors is based on an oscillating crystal, which converts changes in natural frequency into changes in the mass of the analytes for detecting food freshness. The high-sensitivity and real-time detection of these changes are achieved via the resonance frequency shift in piezoelectric crystals [66]. Tolentino et al. [57] developed a piezoelectric sensor based on a silver(I)/polymer composite for detecting ethylene specifically, offering a novel strategy for quality monitoring during postharvest storage and transportation. The working range of this sensor is from 1 to 7 ppm (R = 0.9896), with an RSD of <15% (*n* = 3), with the relative standard deviation of repeatability being <5% (*n* = 11) against 25% ethylene, and the LOD was 420 ppb (Table 2). A trimethylamine (TMA) vapor probe based on a piezoelectric quartz crystal is fabricated for detecting fish spoilage, which is rapid and completely reversible at normal temperature and relatively high humidity. The concentration range of this probe is 5–200 ppm, and the relative standard deviations were less than 5% under 50 ppm TMA within 6 months [58]. Furthermore, the piezoelectric vibration sensor technique is established for assessing the dry matter, which is closely related to potato hardness/freshness, by amplifying the vibration of the surface and converting it into digital form. The amplitude showed quite significant changes in different potato samples, including firm quality potatoes, less firm quality potatoes, and extensively less firm (low dry matter) [59]. The advantages of the piezoelectric biosensors are simplicity, label-free detection, and real-time monitoring, while the sensitivity and specificity are limited.

The above piezoelectric sensor techniques highlight the practical potential for industrial deployment. However, several inherent platform limitations persist, such as the absence of real-time monitoring capability and external validation. These common limitations suggest that, while piezoelectric sensor technology offers a non-destructive approach for monitoring food freshness, its transition from laboratory research to industrial practice requires further improvements in environmental robustness, continuous operation, and multi-parameter detection capabilities. Meanwhile, the integration with artificial intelligence and machine learning is necessary to process complex signals derived from piezoelectric vibration, and more robust prediction models can be established to improve the accuracy and universality of physical and electrical sensing methods.

### 3.3. Electrochemical Biosensors

Electrochemical biosensors are regarded as highly sensitive for detecting indicative analytes, such as biogenic amines and hypoxanthine (HX), in assessing food freshness, relying on recognition elements immobilized on the electrode surface [67]. The specific interactions between the recognition elements and target analytes directly or indirectly generate electrical signals, e.g., changes in current or potential, that are proportional to the analyte concentration [68].

For example, xanthine oxidase (XOD) is immobilized on nanocellulose for the quantitative detection of xanthine (XA) in fish spoilage monitoring, in which the active pH of the biosensor was 7.5, and the linear detection range of XA was 3–50 µM with an LOD of 47.96 nM. The sensitivity of this nanocellulose electrochemical biosensor is 5281 µA mM^−1^ cm^−2^, and the stability is one month with 97% activity (Table 2) [60]. Subsequently, XOD is immobilized onto a copper-based metal–organic framework (Cu-MOF) thin film for detecting HX and XA in chilled seafood freshness, possessing high sensitivity with a wide linear range (0.01 to 10 μM) and good reusability (up to 100 times) [61]. Song et al. [62] developed a 2D layered double hydroxide-based gallium–copper dual single-atom nanozymes platform for the detection of volatile amines. The platform presents a rapid response time of 4 s and achieves an LOD of 5.9 µm, with a good linear relationship between the ammonia concentration and steady-state current over the range of 0.05–0.4 mM (Figure 4B). This platform allowed rapid detection and real-time monitoring of livestock and poultry meat freshness both at room temperature and under refrigerated conditions. Meanwhile, a two-dimensional Cu-MOF/graphene heterojunction electrochemical sensor is proposed to achieve multiple-target detection of fish freshness, including XA, HX, and malachite green (MG). The LOD and corresponding linear ranges of XA, HXA, and MG are 2.9 nM (0.01–5.0 μM), 5.2 nM (0.01–5.0 μM), and 3.1 nM (0.02–2.0 μM), respectively. The results were consistent with those obtained by liquid chromatography-mass spectrometry [63]. A cerium dioxide composite platinum and polymer 3-amino-5-mercapto-1,2,4-triazole (CeO_2_@Pt/triazole) composite sensor is designed to detect XA for evaluating fish freshness. In a pH 6 phosphoric acid buffer solution, this composite sensor exhibits good linear relationships with concentration, with R^2^ = 0.994 (10–138 µmol/L concentration range) and R^2^ = 0.992 (139–330 µmol/L concentration range), accompanied by an LOD of 1.036 µM [64]. In addition, a porous-electrode-capped organic gas sensor system is mentioned to rapidly detect various amine gases from raw fish meat within 1 min, which presented ppb-regime sensitivity compared with the traditional titration method, characterized by the effective ammonia concentration of 100 ppb vs. 18 mg/100 g and 200–300 ppb vs. 28–35 mg/100 g [65].

Driven by material innovation, multi-indicator detection, and device intelligence, electrochemical sensing technology is transitioning from single enzymatic reactions toward real-time and full-chain intelligent food freshness monitoring systems. Although these electrochemical sensor techniques demonstrate relatively low LODs, critical gaps remain, including the lack of external validation, reusability data, matrix interference, and environmental factor challenges (temperature fluctuations, humidity). These limitations highlight that while significant progress has been made, the transition from laboratory results to real industrial deployment requires further validation.

### 3.4. Enzyme-Based Biosensors

Enzyme-based biosensors detect target analytes by utilizing the high specificity and catalytic power of enzymes, which translate biochemical recognition events into measurable physical or chemical signals [69]. Compared to traditional high-performance liquid chromatography or gas chromatography, enzyme-based biosensors serve as simple and rapid tools for evaluating food freshness [70]. Generally, enzyme-based biosensors can be mainly classified into two categories according to the characteristics of their recognition elements.

**Figure 5:**
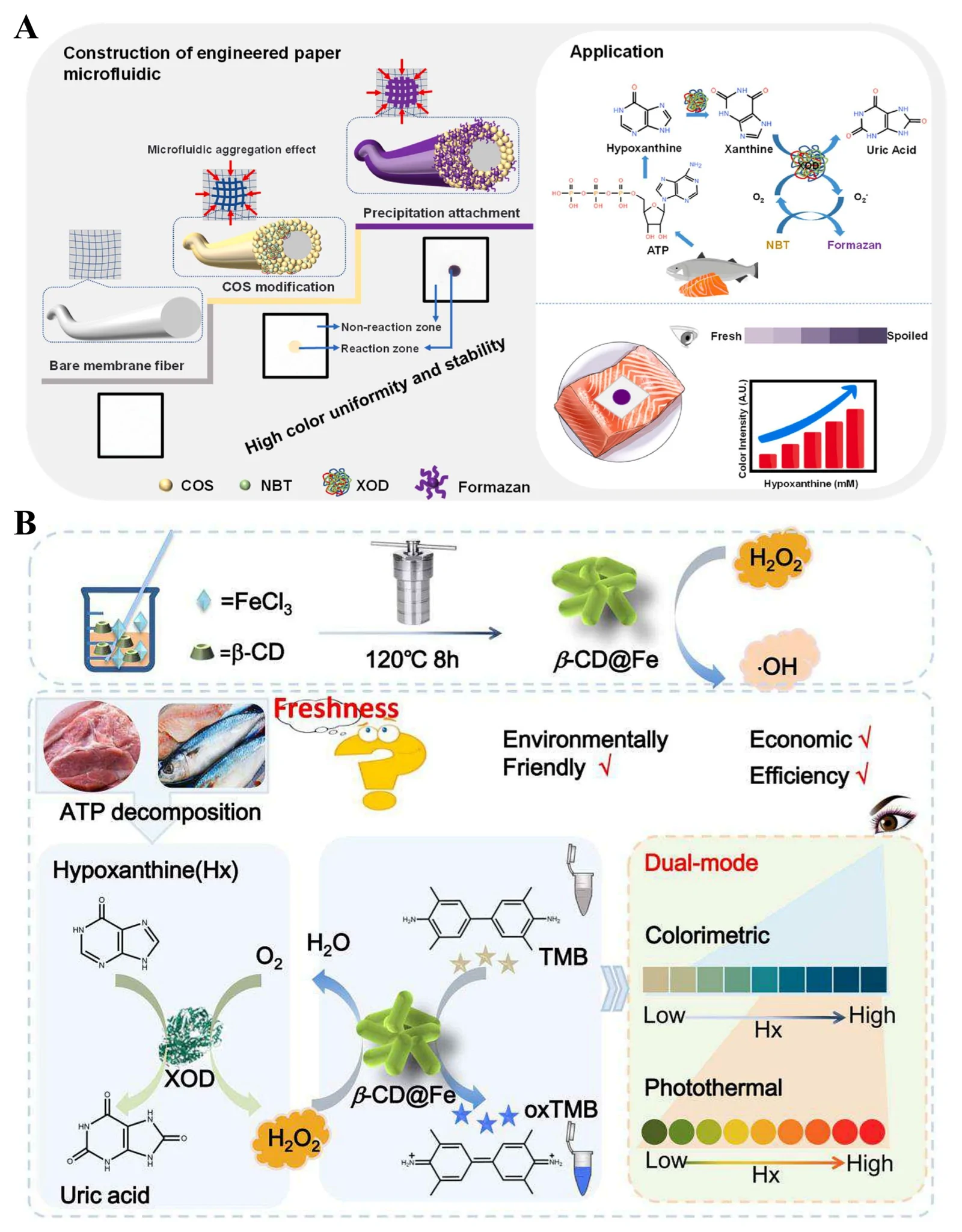
(**A**) Schematic diagram of an integrated HX enzyme sensor with engineered paper microfluidics. Adapted with permission from Ref. [71]. Copyright2024, Elsevier. (**B**) Schematic diagram of β-CD@Fe composite preparation and construction of a dual-mode colorimetric and photothermal visual sensing platform for HX detection used in meat freshness assessment. Adapted with permission from Ref. [75]. Copyright2025, Elsevier.

One is natural enzyme-based biosensors. These sensors directly employ natural enzymes as biological recognition elements to catalyze reactions with spoilage markers (e.g., HX, XA). For example, Wang et al. [71] constructed an integrated HX enzyme sensor based on a chito-oligosaccharide-modified nitrocellulose membrane, in which the electrostatic attraction between chito-oligosaccharide and XOD and the formazan product enhances the enzyme storage stability and the color homogeneity (Figure 5A), achieving 2.30 μM LOD with the linear range 0.05–0.35 mM, with the entire detection process completed in only 5 min (Table 3). This natural enzyme-based biosensor shows high specificity and can be used directly for HX in fish and shrimp samples and different broths.

**Table 3:** Summary of biosensors classified by recognition elements for food freshness monitoring.

Sensor Technology	Food Matrix	Analytes	Response Time	Accuracy	R^2^/Precision	Repeatability/Reproducibility/Robustness	Chemometric Methods	LOD/LOQ	Matrix Interference	External Validation	Cost	Destructive	Ref.
Enzyme-based biosensor	Fish and shrimp	HX	5 min	/	/	High specificity; enhanced enzyme storage stability and color homogeneity via COS electrostatic attraction	/	LOD: 2.30 μM (linear range: 0.05–0.35 mM)	Partial matrix interference from fish/shrimp protein; mitigated by COS modification	Real fish-shrimp complex sample applicability verified	Economical, eco-friendly, suitable for on-site rapid testing via PubMed	Yes	[71]
	Fish	XA and HX	/	/	Good linear fitting for calibration curve	Good anti-interference for fish-sample matrix; acceptable electrode-to-electrode reproducibility	Homemade computer-controlled laser scribing machine	LOD: XT 0.26 μM (linear range 0.3–179.9 μM); HX 0.18 μM (linear range 0.3–159.9 μM)	Minor interference from fish tissue extracts	Compared with classic spectroscopic reference method	Low-cost one-step laser fabrication	Yes	[72]
	MCF-7 cell lysates	ATP	/	/	Good linear response within calibration range	High selectivity against potential co-existing interferences	/	Linear range: 2.0–80.0 μmol/L	/	/	/	Yes	[73]
	Shrimp	HX	/	High accuracy in real sample detection	/	Good robustness against matrix interference; stable signal output in actual shrimp samples	Smartphone-based intelligent analysis	LOD: 9.67 μM (linear range: 18.75–600 μM)	Shrimp protein interference partially suppressed by dual-enzyme system	Real-world shrimp freshness	Economically viable for field detection	Yes	[74]
	Meat	HX	/	Satisfactory spike recovery for meat extracts	Good linear correlation for dual-mode calibration	High anti-interference ability towards meat-matrix co-existing substances	/	LOD: 0.29 μM (colorimetric) and 1.0 μM (photothermal) with linear range: 1–40 μM	Lipid-protein interference in meat matrix	Successfully applied to actual meat samples	Mild-synthesis raw material, cost-effective nanozyme material	Yes	[75]
	Shrimp and mackerel	Histamine	/	Recovery: 94.64–110.65%	RSD < 6%	Acceptable stability under varied storage-condition matrix background	/	LOD: 13.16 μM; LOQ: 18.49 μM	Minor matrix effect from seafood extracts	Tested with shrimp and mackerel under diverse storage conditions	/	Yes	[76]
Antibody-based biosensors	Buffer only (no real-food samples)	Histamine	Assay runtime ~2–3.25 h (pre-processing excluded)	/	/	/	Flatbed-scanner acquisition → grayscale conversion → spot-signal extraction	LOD (oCNP): 6–8 μg/mL; LOQ unreported	/	Not provided	Carbon-black raw material cost-effective	Yes	[77]
	Salmon and wine	Histamine	Antibody-based 120 min, aptamer-based 80 min; instrument readout 5 s per sample	Antibody assay: fish 103–107%, wine 95–105%	Fish: antibody 3–11%, aptamer 7–16%; wine: antibody 6–11%, aptamer 3–12%	Low cross-reactivity (<0.1%)	Time-gated FRET measurement with non-linear fitting.	Antibody-based LOD = 0.19 μg/mL, LOQ absent	/	Conventional heterogeneous ELISA method.	Needs dedicated time-gated fluorescence plate reader.	Yes	[78]
	Buffer only (no real-food samples)	Histamine	Incubation 3 h 15 min (chip spotting and readout excluded).	No real-sample spike-recovery	CV: 4–17%	Probes stable for 5-month storage	Microarray scanner-GenePix-Origin analysis.	/	/	/	Depends on commercial antibodies	Yes	[79]
	Fresh and expired/spoiled ready-to-eat foods	Lactic acid bacteria (LAB)	Assay runtime ~1 h (enrichment excluded)	Spoilage detection accuracy 82% overall; poorer performance (63%) for meat due to matrix interference.	/	Matrix interference in meat sample.	LFIA: strip grayscale captured by scanner, manually scored	LFIA LOD: 4–5 log_10_ cfu/test; Acid-producing pH-test LOD: 6–7 log_10_ cfu/test	/	Validated by microbiota sequencing and MRS-agar plate counting	LFIA requires no bulky instruments yet needs pH hardware	Yes	[80]
Nucleic acid-based aptasensors	Oyster meat	ATP	Assay runtime 70 min (pre-treatment excluded)	Spike recovery 92.73–107.14%	R^2^ = 0.99; intra-/inter-day RSD < 5%	Good selectivity; inter-batch probe reproducibility untested	UV-Vis spectrophotometer measurement with linear fitting.	LOD = 0.065 μM	/	Correlated with TVC reference	No large-scale instruments needed	Yes	[81]
	Perch and shrimp meat	ATP	Assay runtime 70 min (pre-treatment excluded)	/	R^2^ = 0.990–0.999 for ATP-TVC correlation	Satisfactory selectivity; inter-batch and long-term stability data absent	Dual-mode UV-Vis-fluorometer detection	Colorimetric LOD = 71 nM; fluorescent LOD = 73 nM	/	TVC as reference method	Instrument-friendly	Yes	[82]
	Hairtail and leatherjacket	Histamine	Reaction finished within 40 min (pre-treatment excluded)	Spike recovery 82.24–105.92%	Dual-mode R^2^ = 0.9996. Low RSD	Inter-batch and long-term stability unevaluated	Dual-mode fluorometer-UV-Vis detection	Combined-signal LOD = 4.57 nmol/L	/	Consistent with national-standard HPLC	No monetary cost values given	Yes	[83]
	Salmon and shrimp	Histamine	Assay runtime 45 min (filtration-centrifugation excluded)	Results correlated with commercial ELISA kits	R^2^ = 0.9911	Good selectivity	UV-Vis detection with logarithmic fitting	LOD = 1.89 nM	/	Validated against ELISA	No monetary cost values given	Yes	[84]
	Buffer, cell medium, mammalian cells (no food samples)	ATP, thrombin	ID-HCR incubation 120 min	Experiments are qualitative/semi-quantitative	/	Satisfactory selectivity	Fluorometer/confocal measurement and Origin fitting.	ATP: LOD = 2.1 nM; Thrombin: LOD = 3.7 pM d	/	Not performed	No monetary-cost values given	Yes	[85]
Metabolite-indicating sensors for assessing microbial spoilage	Beef	Volatile ammonia	No fixed response time	/	R^2^ = 0.9067;	Within-batch color consistency; inter-batch data missing.	Image-acquired CIE-Lab ΔE calculation.	/	/	Correlated with national-standard TVB-N	No monetary-cost values given	No	[86]
	Meat	ATP	Reaction 120 min	Readouts agreed with commercial ATP kits; ATP-TVC correlation confirmed	/	/	Glucometer-based linear calibration	LOD = 1.08 nmol/L	/	Referenced to ATP kit and TVC plate counting	No monetary-cost values given	No	[87]
	Oyster	Mixed food VOCs)	Several minutes; sample storage-aging time excluded	Test duration: several minutes (sample aging excluded)	/	/	PCA chemometric analysis	Only sample classification, no single-analyte quantification	/	Benchmarked against GC-MS, TVB-N, plate counting and sensory evaluation	No monetary-cost values given	No	[88]

Due to the difficulties in preparing and purifying natural enzymes, artificial enzymes nanozymes have been designed to be used as a substitute. Nanozymes typically integrate catalytic performance with unique optical or electrical properties, thereby facilitating the development of portable and multifunctional sensing devices. Regarding nanozyme-based sensors, Zhu et al. [72] prepare a flexible 3D porous graphene nanozyme electrode on polyimide via direct laser-writing technology and evaluate fish freshness via simultaneous detection of both XA and HX, followed by smart analysis and digital output using a homemade computer-controlled laser scribing machine coupled with ANN algorithms. The LODs of XA and HX are 0.26 μM (with a linear range 0.3–179.9 μM) and 0.18 μM (0.3–159.9 μM), respectively. A novel “Off–On” colorimetric sensing system based on synthesized quercetin@ZIF-90 and MnO_2_ nanosheets is designed for detecting adenosine triphosphate (ATP), whose concentration variation indicates cellular activity. Owing to the strong antioxidant and radical scavenger activity of Quercetin@ZIF-90, this colorimetric sensor exhibits a good linear range from 2.0 μmol/L to 80.0 μmol/L under laboratory conditions [73]. Dong et al. [74] construct a colorimetric nanozyme sensor based on a dual-enzyme system consisting of Ni-Pt nanozymes and natural XOD; it realized high practical robustness in monitoring real shrimp samples, characterized by a linear detection range of 18.75 to 600 μM and an LOD of 9.67 μM. Additionally, β-cyclodextrin-reduced Fe(III) nanozyme (β-CD@Fe) is synthesized for the colorimetric and photothermal dual-mode detection of HX in meat freshness (Figure 5B), along with a good linear relationship in the range of 1–40 μM and low LODs: 0.29 μM for colorimetric detection and 1.0 μM for photothermal detection, respectively [75]. Meanwhile, a multicolor determination system for histamine is designed based on the CDsEDTA@Fe^3+^ nanozyme for rapid monitoring of shrimp and mackerel freshness under different storage conditions, and the LOD and quantification limits are 13.16 μM and 18.49 μM, respectively [76].

The current development of enzyme-based sensors for food freshness detection is shifting from reliance on highly specific natural enzymes toward the use of multifunctional and highly stable nanozymes. The technology readiness landscape across these enzyme-based sensors varies considerably. Among them, the dual-enzyme system constructed by Dong et al. is close to commercial deployment [74], and the flexible 3D porous graphene nanozyme electrode prepared by Zhu et al. offers attractive manufacturing scalability [72]. Meanwhile, the primary challenge in constructing enzyme-based biosensors remains enzyme stability and feasibility under real conditions. Moreover, comprehensive cost analyses are essential for further commercialization. Future efforts should prioritize field validation and systematic life-cycle cost modeling to bridge the remaining gap between laboratory results and industrial adoption. Importantly, the integration of enzyme-based sensors with flexible electronics, smartphone terminals, and AI algorithms is driving the evolution of detection technologies toward greater intelligence, integration, and visualization.

### 3.5. Antibody-Based Biosensors

The core principle of antibody-based biosensors lies in the highly selective recognition of specific markers generated during food spoilage (e.g., histamine, spoilage microorganisms) via specific antigen–antibody binding. Using immunoassay strategies such as competitive or sandwich formats, these microscopic chemical or biological signals are converted into intuitive physical signals, including color and fluorescence, enabling rapid and sensitive assessment of food freshness. This approach possesses high specificity and operational simplicity, making it particularly suitable for on-site rapid screening and quality control.

**Figure 6:**
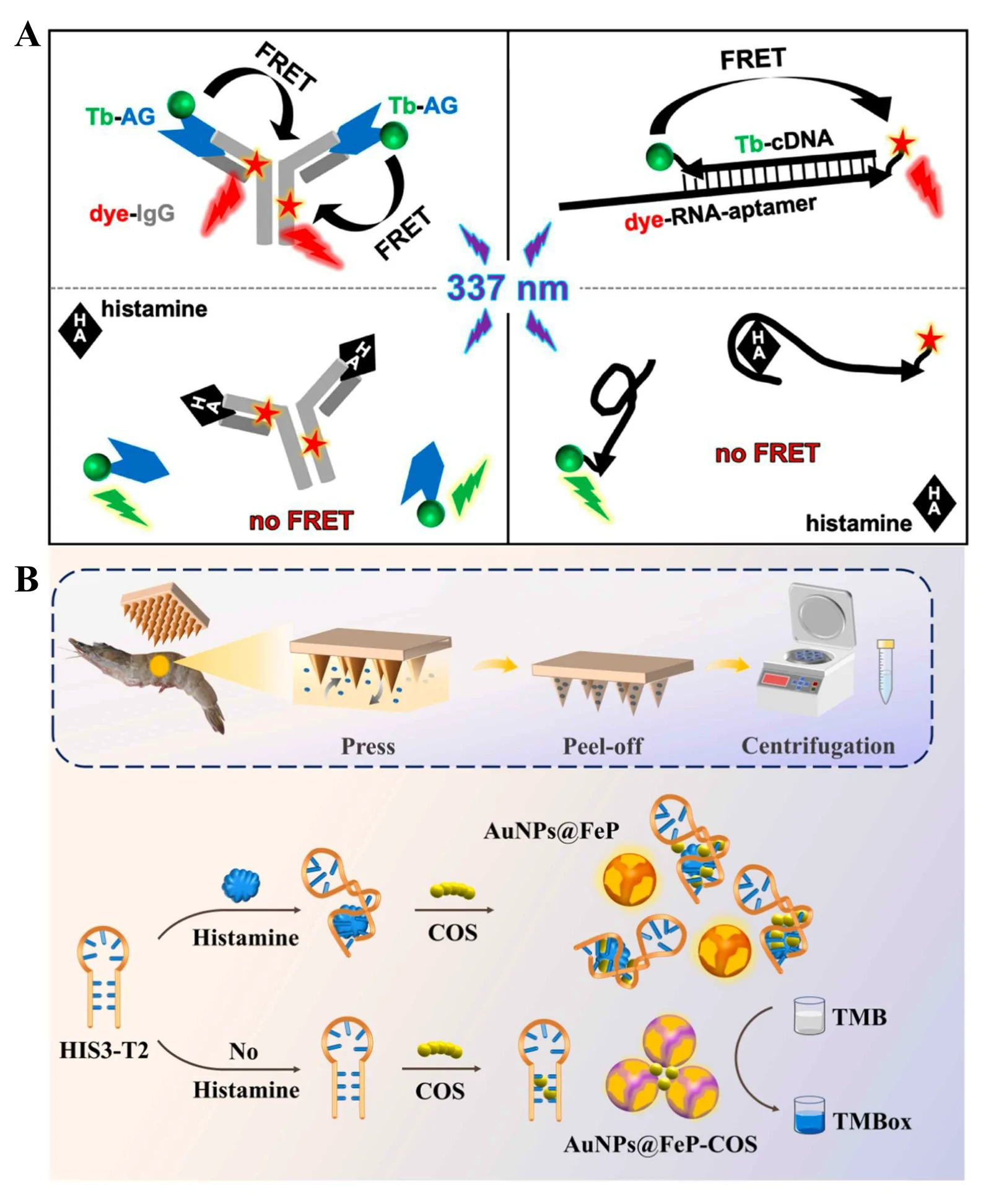
(**A**) Detection principle of TG-FRET immunosensor. Adapted with permission from Ref. [78]. Copyright 2022, American Chemical Society. (**B**) Schematic diagram of an integrated colorimetric aptasensing platform for histamine extraction and analysis in seafood. Adapted with permission from Ref. [84]. Copyright 2025, Elsevier.

Based on the antibody competitive inhibition principle, Mattsson et al. developed a microarray chip detection system by modifying the availability of carbon black nanoparticles as colorimetric immunoassay labels (Table 3) [77]. This system targets histamine in food samples with performance comparable to that of fluorescent labeling. Subsequently, a time-gated Förster resonance energy transfer (TG-FRET) immunosensor was constructed based on competitive binding between antibodies and terbium-labeled antigens, realizing rapid and low-cost detection of histamine in fish and wine (Figure 6A) [78]. Then, a fluorescence chip immunoassay method is established using a commercial polyclonal antibody (H7403, Sigma-Aldrich), followed by a competitive inhibition immuno-chip system with histamine detection at a dynamic range of 8–111 µg/mL [79]. In addition, based on *Lactobacillus plantarum* antibodies, a lateral flow immunochromatographic assay strip was developed by combining it with a semiconductor pH sensor; it can monitor acid production capacity and realize rapid on-site screening of food spoilage caused by lactic acid bacteria [80]. Antibody-based biosensors exhibit remarkable advantages for rapid food freshness screening owing to their high specificity and convenience. It has evolved into various sensitive detection platforms, including microarray chips and time-gated fluorescence sensors. Nevertheless, antibody-based biosensors suffer from inherent drawbacks, including poor antibody stability, high production cost, reduced activity after immobilization, and batch-to-batch variation, while real food testing and supply chain trials are still insufficient. Thus, future efforts should focus on optimizing anti-matrix interference capacity, completing multi-level validation from real sample assessment to supply chain pilot tests, as well as AI-aided recognition-molecule design to achieve stable, manufacturable detection technologies.

### 3.6. Nucleic Acid-Based Aptasensors

Aptamers are short oligonucleotide sequences obtained through in vitro selection, which can bind specifically and efficiently to corresponding ligands (e.g., proteins, small biomolecules, biotoxins, and other metabolites) [89]. Accordingly, aptamers can serve as recognition elements within DNA nanostructures for specific molecular recognition, forming aptasensors. Nowadays, ATP biosensing strategies based on hemin/G-quadruplex (G4) DNAzymes have emerged as promising methods and have attracted extensive attention. The core basis is that the specific recognition of ATP by its aptamer triggers the formation of a G-quadruplex structure, which further combines with hemin to yield a DNAzyme with peroxidase-like activity. By monitoring the signals generated by the catalytic activity of the DNAzyme, quantitative analysis of ATP can be achieved in evaluating the freshness of aquatic products.

For instance, Dai et al. developed an isothermal ATP-targeting aptasensor, enabling reliable evaluation of oyster freshness (Table 3 ) [81]. After that, Si et al. designed a robust ATP sensing system using a dual-mode hemin-G4 DNAzyme, which is successfully applied to assess seabass freshness under various storage conditions [82]. Furthermore, a dual-mode detection method was proposed by integrating aptamer recognition and time-resolved fluorescence resonance energy transfer, realizing rapid, accurate, and visual detection of histamine in fish samples [83]. Meanwhile, an integrated colorimetric biosensing platform was constructed consisting of a poly(vinyl alcohol)/HA microneedle patch and an aptasensor of AuNPs@FeP-chitosan oligosaccharide (COS) nanozymes. This system realized highly sensitive and selective detection of histamine in real salmon and shrimp samples (Figure 6B) [84]. Gao et al. [85] developed an enzyme-free autocatalytic feedback DNA circuit sensor by integrating DNAzyme and hybridization chain reaction. This sensor achieves signal amplification through DNA self-assembled nanostructures and is used for the highly sensitive detection of ATP and thrombin. Therefore, aptamer-based sensing strategies provide sensitive, convenient, and reliable rapid detection of aquatic product freshness through the rational integration of specific recognition, signal amplification, and multi-mode signal output.

Current aptasensors mostly achieve ideal performance under buffer-based laboratory proof-of-concept conditions, while poor stability in real-food matrices and the absence of portable-system validation and industry-oriented standardization limit their translation out of the laboratory. Further work should focus on improving anti-interference performance for complex food samples, conducting successive real-food testing, portable-system verification, and supply chain trials to push forward standardization and industrial deployment.

### 3.7. Metabolite-Indicating Sensors for Assessing Microbial Spoilage

During food spoilage, microbial proliferation and metabolism are key drivers of food quality deterioration. Instead of capturing microbial cells directly, metabolite-indicating sensors assess food freshness indirectly by measuring metabolic products generated by microbial activity. Through integration with advanced functional materials and signal-amplification strategies, these sensors achieve improved monitoring performance and practicability in complex food matrices. Two technical development routes can be summarized for such metabolite-indicating platforms: “on-site rapid detection” and “laboratory high-precision detection.” The former delivers field-monitoring capacity via intuitive color readout and portable devices; the latter enables ultrasensitive quantification and visualization of microbial metabolic signatures. Combining these two routes with novel preservation technologies forms a multi-level evaluation system covering from macroscopic food quality judgment down to microscopic metabolic profiling of food spoilage processes.

**Figure 7:**
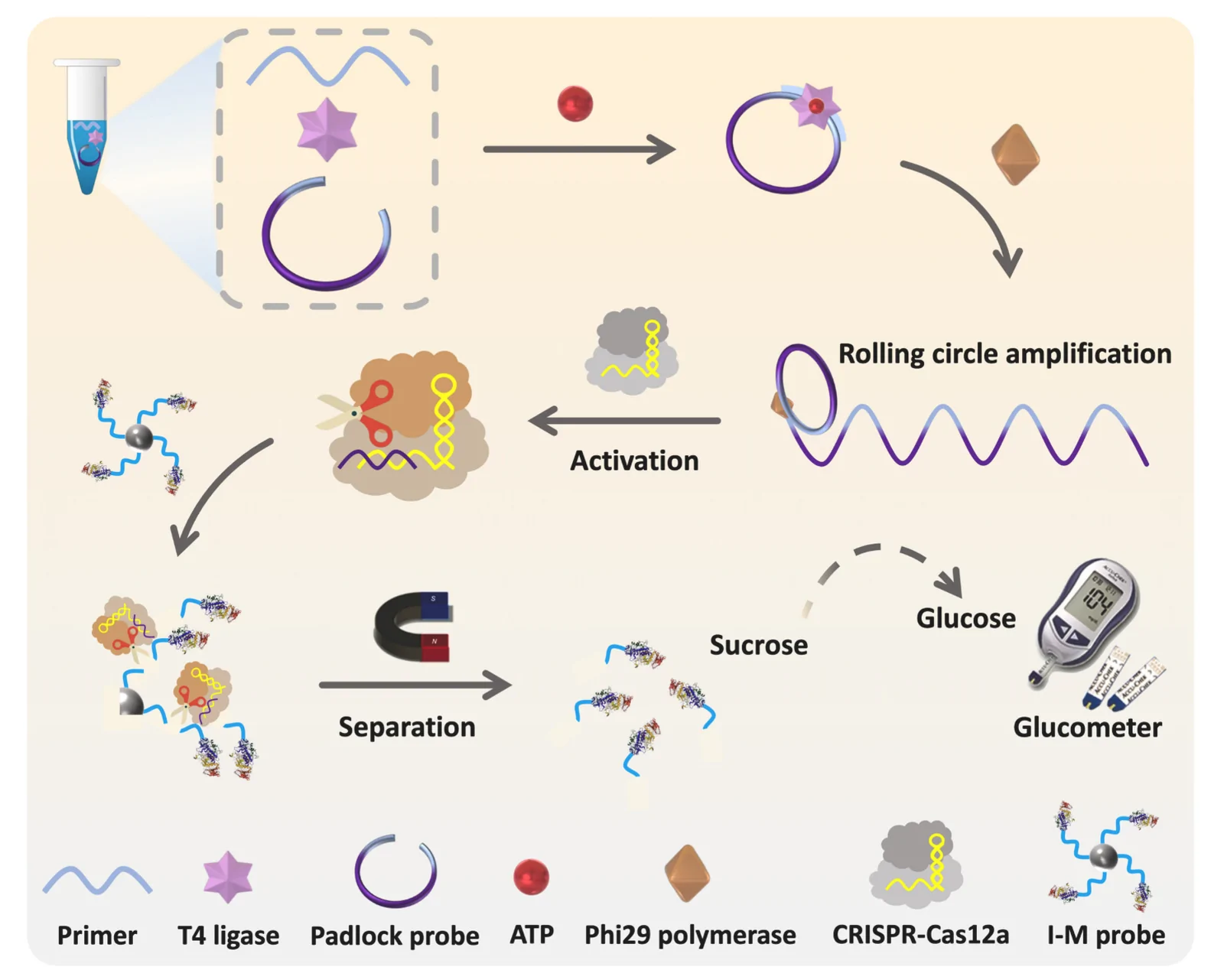
Schematic diagram of the strategy for ATP detection based on a personal glucometer using cofactor-dependent enzymatic ligation-triggered RCA-assisted CRISPR-Cas12a. Adapted with permission from Ref. [87]. Copyright 2025, Elsevier.

For meat samples, an ammonia-responsive colorimetric microbial sensor film was designed based on alizarin encapsulated in ZIF-8 (AL@ZIF-8); it can indirectly monitor beef freshness by detecting microbial metabolites (i.e., ammonia and amines, Table 3) [86]. Then, cofactor-dependent enzymatic ligation-triggered rolling circle amplification (RCA) coupled with CRISPR-Cas12a is designed for quantitative detection of ATP on the surfaces of lamb and beef (Figure 7) [87]. The significant correlation between ATP and TVC shows the feasibility for rapid meat freshness assessment. With regard to aquatic products, a gas sensor system was constructed based on ten metal oxides and acts as a sensor array to detect volatile gases (e.g., ammonia, amines, sulfides) generated from oyster spoilage under different storage temperatures [88]. In fact, current microbial sensor development includes two pathways: “on-site rapid detection” and “laboratory high-precision detection.” The former achieves on-site monitoring through intuitive color changes and portable devices, while the latter realizes ultrasensitive quantification and visualization of cell metabolism. The combination of these two pathways constructs a multi-level assessment system for food freshness and biological processes when combined with novel preservation technologies, ranging from macroscopic quality judgment to microscopic metabolic monitoring. However, most metabolite-indicating sensors are validated only via laboratory proof-of-concept experiments; challenges remain in real-food anti-interference, portable-system performance calibration, supply chain trials, and unified standardization for industrial deployment. Subsequent studies need to optimize material selectivity for complex food matrices and carry out progressive validation, including real food testing, portable-system assessment, and supply chain trials to facilitate standardized industrial application.

## 4. Rapid Detection Technologies Based on Spectroscopy and Imaging

### 4.1. Near-Infrared Spectroscopy (NIR)

**Figure 8:**
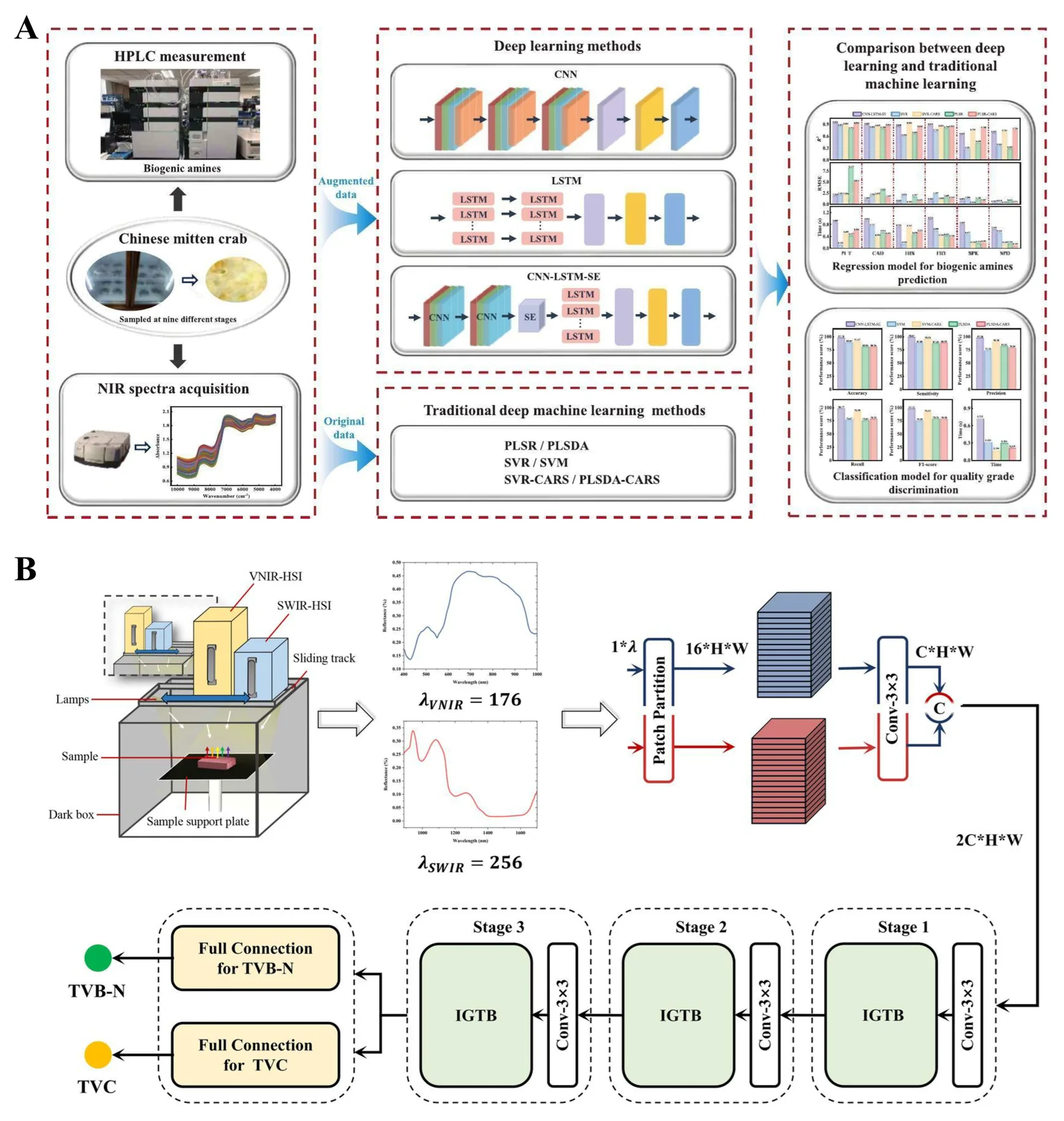
(**A**) Flowchart of a NIR hybrid deep-learning model for assessing Chinese mitten crab freshness. Adapted with permission from Ref. [94]. Copyright 2025, Elsevier. (**B**) Flowchart of a Hyperspectral Imaging hybrid multi-task interleaved group transformer model for assessing food freshness. Adapted with permission from Ref. [95]. Copyright 2025, Elsevier.

NIR is a non-destructive analytical technique grounded in molecular vibrational spectroscopy. It relies on the absorption and scattering of specific near-infrared radiation by hydrogen-containing functional groups within samples, including moisture, fat, and protein. It is extensively applied in the food industry owing to its speed and precision. For pork, Zhang et al. achieve robust prediction of TVB-N content by using a dual-band sensor based on just-in-time learning (JITL), with a coefficient of determination R^2^ of 0.95 and a root mean square error of prediction (RMSEP) of 1.60 mg/100 g (Table 4) [90]. Sun et al. established a high-precision prediction model for evaluating TVB-N by combining visible-near-infrared two-dimensional correlation spectroscopy images with a dual-branch CNN, and the model achieved high accuracy (R^2^*p* = 0.9579 and RMSEP = 0.8093 mg/100 g) [91]. In addition, Zhao et al. [92] developed an integrated machine learning (ML)–deep-learning (DL) modeling strategy, whereby hybrid convolutional neural network–partial least squares regression (CNN-PLSR) and convolutional neural network–support vector regression (CNN-SVR) models were built for pork freshness evaluation. The model achieved high accuracy in predicting pork pH and TVB-N content with R^2^ values of 0.9253 and 0.9342, respectively [92]. For aquatic products, a mid-level data fusion strategy based on Raman and NIR spectral data significantly improved the estimation performance of TVB-N levels in Pacific white shrimp via a portable spectrometer [93]. The quantitative prediction of six biogenic amines and the quality grading of Chinese mitten crab (*Eriocheir sinensis*) are realized by a hybrid convolutional neural network–long short-term memory-squeeze-and-excitation (CNNLSTM-SE) model (R^2^ > 0.84, RMSE < 2.0) (Figure 8A) [94].

**Table 4:** Summary of spectroscopy and imaging technology applications in food freshness monitoring.

Sensor Technology	Food Matrix	Analytes	Methods	Response Time	Accuracy	R^2^/Precision	Repeatability/Reproducibility/Robustness	LOD /LOQ	External Validation	Destructive	Ref.
Near-Infrared Spectroscopy	Pork	TVB-N	JITL-LSSVM model	/	Root mean squared error of prediction set (RMSEP) =1.60 mg/100 g	R^2^*p* = 0.95	Ratio of performance deviation (RPD) = 4.24	/	Yes	No	[90]
	Pork	TVB-N	CNN with vis-NIR 2D-COS images	/	RMSEP = 0.8093 mg/100 g	R^2^*p* = 0.9579	RPD = 4.5769	/	Yes	No	[91]
	Pork	pH value and TVB-N	CNN-PLSR and CNN-SVR hybrid deep-learning models	/	pH value RMSEP = 0.0731, TVB-N RMSEP = 2.3963	pH value R^2^p =0.9253, RPD = 3.619, TVB-N R^2^*p* = 0.9342	pH value RPD = 3.619, TVB-N RPD = 4.095	pH value (LOD = 0.25, LOQ = 0.77) and TVB-N (LOD = 7.84, LOQ = 23.74)	Yes	No	[92]
	Pacific white shrimp	TVB-N	CNN-ELM-BPC prediction model	/	RMSEP = 0.677 mg/100 g	R^2^*p* = 0.986	/	/	Yes	No	[93]
	Chinese mitten crabs	Six biogenic amines (BAs)	CNN-LSTM-SE hybrid model	/	RMSE < 2.0	R^2^ > 0.84	/	/	Yes	No	[94]
Hyperspectral Imaging	Spinach and Chinese cabbage stored	Average spectra	CNN-LSTM model	/	Accuracy > 80%	/	/	/	No	No	[96]
	Red meat	Moisture content	PLSR, LS-SVM, and MLR multivariate calibration models	/	RMSEP (%) = 2.19	R^2^*p* = 0.97	RPD = 3.619	/	No	No	[97]
	Beef	The pH and the color indices L* and b*	PLSR and LSSVM models	/	pH RMSEP = 0.0905, L* RMSEP = 1.0914, b* RMSEP = 0.7498	pH R^2^*p* = 0.7440, L* R^2^*p* = 0.7941, b* R^2^*p* = 0.8781	/	/	No	No	[98]
	Chicken breasts	TVB-N and total viable count (TVC) contents	Multi-task interleaved group transformer model (MIGTM)	/	TVB-N RMSEP = 2.1988, TVC RMSEP = 0.1483	TVB-N R^2^V = 0.9040, TVC R^2^V = 0.9499	/	/	No	No	[95]
	Lamb meat	TVB-N, TVC, pH, and L*		/	TVB-N RMSEP = 2.28, TVCRMSEP = 0.84, pH RMSEP = 0.19, L* RMSEP = 1.83	TVB-N R^2^*p* = 0.93, TVC R^2^*p* = 0.91, pH R^2^*p* = 0.89, L* R^2^*p* = 0.88	TVB-N RPD = 2.99, TVC RPD = 4.70, pH RPD = 2.45, L* RPD = 1.89	/	Yes	No	[99]
Raman Spectroscopy	*Lateolabrax* *japonicus*	TVB-N determination and SDS-PAGE pattern	Sensor	0.44 s	90.6%	/	/	/	Yes	Yes	[100]
	Salmon flesh	Thiobarbituric acid reactive substances	CNN	/	RMSEP = 0.103 mg MDA/kg	R^2^*p* = 0.866	RPD = 4.136	/	Yes	No	[101]
	Shrimp	TVB-N and pH	PLSR model	/	/	R^2^*p* = 0.975	RPD = 6.153	/	Yes	/	[102]
Fluorescence Spectroscopy	Frozen pork	TVB-N and pH	PLSR model		TVB-N RMSEP = 3.3202, pH RMSEP = 0.0688	TVB-N R*p* = 0.9151, pH R*p* = 0.9703	TVB-N RPD = 2.28, pH RPD = 3.8	/	Yes	No	[103]
	Freeze–thaw treated frozen-whole-round tilapia	TVB-N	CNN	0.48 s	RMSEP = 1.1502	R^2^*p* = 0.9779	RPD = 6.721	/	Yes	No	[104]
	/	Biogenic amines	/		/	/	/	LOD = 1.41956 × 10^−6^ M	No	/	[105]

NIR spectroscopy for food freshness detection is continuously advancing toward multi-modal fusion and intelligent analysis. Methodologies are gradually shifting from traditional chemometric approaches toward the integration of deep learning and multi-spectral information fusion. By incorporating time-series prediction, multi-source data collaboration, and interpretable model construction, this technique enables not only high-precision quantification of spoilage indicators but also drives systematic progress toward applications in complex food systems, portability, and online real-time monitoring.

### 4.2. Hyperspectral Imaging (HSI)

HSI is an advanced detection technique that can simultaneously acquire both spatial morphological information and continuous spectral data of a target, realizing the integration of imaging and spectroscopy. By collecting reflectance or transmittance data across multiple narrow bands covering the ultraviolet to near-infrared regions and combining them with chemometric methods, HSI allows correlation analysis between the internal components and external characteristics of food products [106]. In the freshness detection of meat and other food matrices, HSI employs spectral features in the visible-near-infrared range to achieve non-destructive and efficient evaluation of both appearance quality and internal chemical variations. This provides a reliable technical foundation for online real-time monitoring.

By combining HSI with a CNN-LSTM deep-learning model, the classification accuracy of spinach and Chinese cabbage freshness exceeds 80%, which significantly improves the utilization efficiency of spectral features (Table 4) [96]. By combining it with a multiple linear regression model, Kamruzzaman et al. achieve the rapid monitoring of moisture content related to the freshness of beef, lamb, and pork [97]. By fusing spectral features extracted from competitive adaptive reweighted sampling and texture features derived from the gray-level co-occurrence matrix, partial least squares regression (PLSR) and least squares support vector machine (LSSVM) models are further developed to predict beef pH and color indices [98]. Regarding chicken breast, a multi-task interleaved group transformer model that fuses dual HSI data is proposed to predict TVB-N and TVC contents simultaneously and precisely, with R^2^*p* values of 0.9040 and 0.9499, respectively (Figure 8B) [95]. For lamb, Zhang et al. constructed a robust assessment model for predicting TVB-N, TVC, and pH based on the relationship between the red shift in the spectral peak at 620–630 nm and the oxidation state of myoglobin [99].

HSI technology trends show that analytical strategies are evolving from sole raw-spectrum utilization toward mid-level spectral-texture data fusion. Algorithms have advanced from conventional chemometrics to deep learning, ensemble learning, and multi-task modeling for simultaneous prediction of multiple freshness attributes. In addition, screening representative feature wavelengths lays theoretical support for developing low-cost multi-spectral online industrial detection systems. Nevertheless, several limitations remain. Most existing experiments are carried out under controlled laboratory conditions, leading to limited cross-species and on-site generalization performance.

### 4.3. Raman Spectroscopy

**Figure 9:**
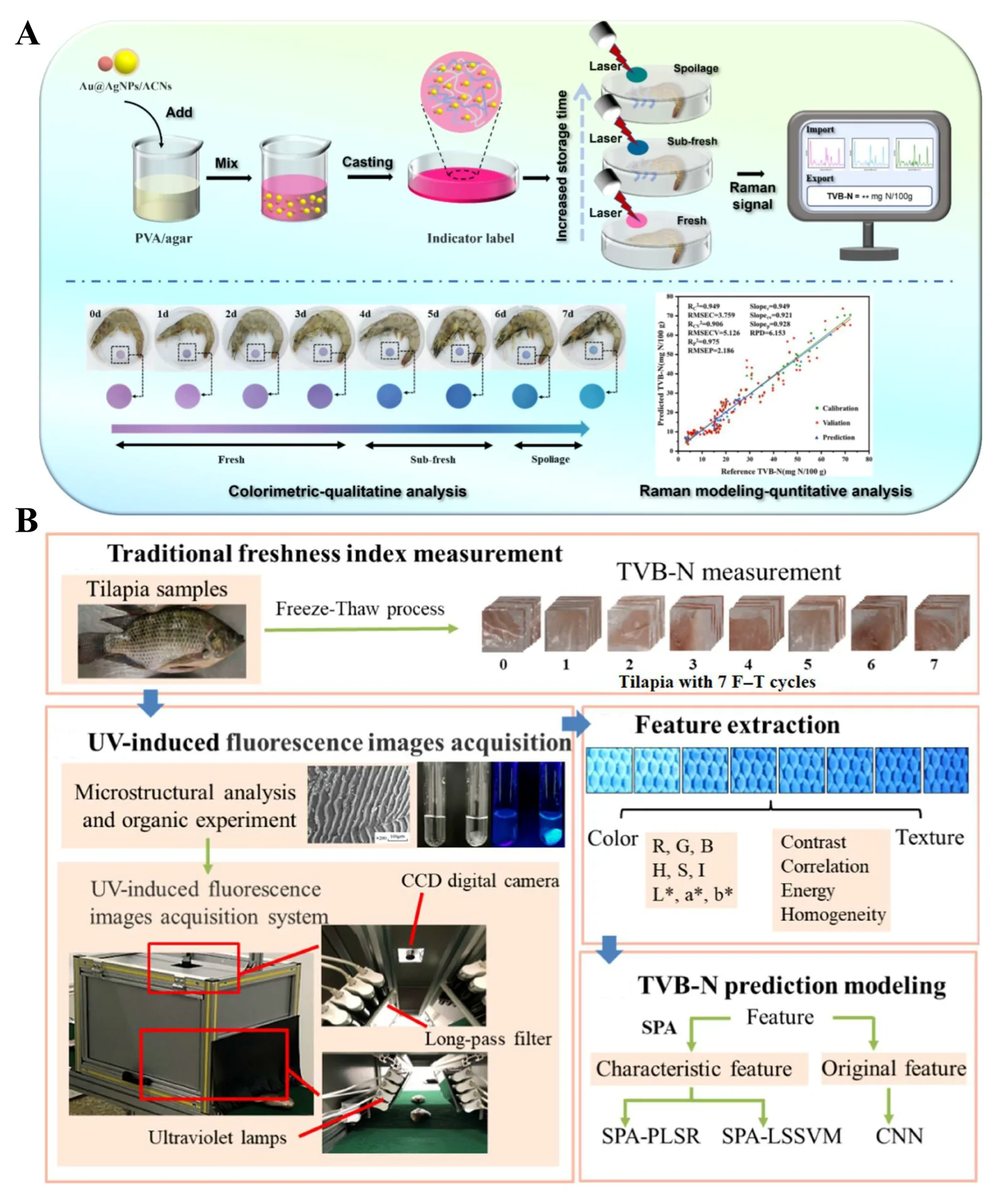
(**A**) Schematic diagram of dual-mode colorimetric and SERS detection of seafood freshness. Adapted with permission from Ref. [102]. Copyright 2025, Elsevier. (**B**) Schematic diagram of UV-induced fluorescence imaging system combined with CNN model for predicting tilapia TVB-N content, L*a*b* represented the stytle of color converter based on CIE LAB model. Adapted with permission from Ref. [104]. Copyright 2024, John Wiley and Sons.

Raman spectroscopy exploits the “fingerprint” effect arising from the interaction between laser radiation and food molecules to monitor variations in specific chemical bonds during spoilage processes, such as protein denaturation and lipid oxidation. It has been developed into a rapid, non-destructive, and highly sensitive modern analytical detection technique. For aquatic products, NIR and Raman spectra are fused to realize accurate prediction of TVB-N in quantitative freshness detection of salmon [107]. Also, Raman spectroscopy is fused with a CNN system to establish a rapid model for sea bass fillet freshness classification, with accuracy reaching 90.6% with a single detection time of only 0.44 s (Table 4) [100]. In addition, a nanostructured surface-enhanced Raman scattering (SERS) sensor enables in situ detection of components in salmon by integrating into a stretchable antimicrobial packaging film, such as purines and proteins [108]. Moreover, NIR-Raman multimodal spectroscopy combined with a CNN model is constructed for quantitative prediction of thiobarbituric acid reactive substances in salmon with R^2^ values of 0.866 [101]. Liu et al. fabricated an intelligent hydrogel patch with both colorimetric and SERS responses, which qualitatively determines shrimp freshness grade via color variation, but also establishes a PLSR model for the quantitative prediction of TVB-N (Figure 9A) [102]. Raman spectroscopy is evolving from a standalone spectroscopic analysis tool toward integrated systems combining deep learning, multispectral data fusion, and smart materials. This trend has significantly enhanced its ability for in situ, real-time monitoring of food spoilage processes. Its application has also expanded from high-precision laboratory detection to portable devices and active packaging. Improving signal sensitivity, optimizing model interpretability, and promoting standardization are crucial for further facilitating widespread practical application throughout the food supply chain.

### 4.4. Fluorescence Spectroscopy

Fluorescence spectroscopy stimulates endogenous fluorophores associated with food freshness to emit fluorescence by irradiating a specific excitation wavelength of the food sample. This technique enables indirect and rapid evaluation of food freshness. Zhuang et al. employed a portable 365 nm UV-LED fluorescence spectroscopy system to realize non-destructive detection of TVB-N and pH values in pork stored at different refrigerated temperatures (Table 4) [103]. Whilst using a UV-induced fluorescence imaging system, Wang et al. extracted multi-color space features successfully and realized high-precision prediction of TVB-N content in tilapia based on a CNN model (Figure 9B) [104]. Additionally, a novel Zn(II) indole Schiff base complex is synthesized to achieve multispectral analysis because of the distinct fluorescence responses to biogenic amines, such as phenethylamine and cadaverine [105]. Indeed, fluorescence spectroscopy approaches for food freshness detection are undergoing significant evolution toward portability, intelligence, and integration with advanced materials. Applications of this technology have expanded from offline laboratory component analysis to on-site rapid identification, real-time dynamic monitoring throughout the supply chain, and the development of novel sensitive materials. Further improvements in detection sensitivity, device standardization, and cost optimization will support its wider adoption in comprehensive intelligent food quality monitoring in the future.

Each of these spectroscopic technologies exhibits distinct strengths and limitations. NIR and HSI enable high-precision quantification of indicators such as TVB-N and pH. SERS offers molecular-level specificity, whereas fluorescence is suited for rapid screening but exhibits limited quantitative capability. In terms of stability, NIR is the most robust, while HSI is susceptible to hardware drift. SERS presents the most challenging signal reproducibility, and fluorescence faces photobleaching risks. Regarding calibration and deployment, NIR features low cost and the highest engineering maturity. HSI entails complex calibration and is more suitable for offline inspection. SERS is well suited for in situ detection but is constrained by instrument cost. Fluorescence enables sub-second portable screening. Overall, multimodal fusion coupled with intelligent modeling represents an inevitable pathway to overcome single-technology bottlenecks and realize intelligent monitoring across the full supply chain.

The neural network architectures in this review are selected based on data modality, task requirements, and deployment constraints. CNN/ResNet34 excel at image feature extraction but require large datasets; YOLOv4-Tiny enables lightweight embedded real-time detection with slightly lower accuracy; CNN-LSTM handles spatiotemporal time-series data; the transformer suits high-dimensional long-sequence multi-task prediction despite high computational cost; customized dual-branch and attention networks address multimodal fusion; transfer learning mitigates small-sample overfitting; and BP networks serve as stable baselines for small datasets. In summary, careful selection of a suitable network architecture must be made in accordance with practical conditions.

## 5. Conclusions and Perspectives

To further facilitate intuitive horizontal comparison among the different technical routes, Table 5 summarizes the core strengths, bottlenecks, application suitability, cost, and technology readiness of the three major categories of freshness-detection approaches. This overview complements the literature-oriented performance data compiled in Table 1, Table 2, Table 3 and Table 4 and offers a high-level perspective for evaluating translational potential. As the comparison indicates, each technical family possesses distinct inherent merits and compromises. Sensory-simulation tools excel at non-destructive, rapid screening and are well suited to industrial sorting and smart-packaging scenarios, yet they are hindered by environmental drift and the difficulty of absolute quantification. Biosensors provide superior molecular specificity for spoilage biomarkers, and the emergence of nanozyme materials represents an important innovation for mitigating the instability of natural enzymes; nevertheless, most biosensor workflows still rely on sample pretreatment and remain constrained by the stability of their sensing elements. Spectroscopic imaging techniques stand out for reagent-free, high-throughput, non-destructive measurement, but their practical deployment is restrained by instrument cost and by calibration-transfer barriers across diverse food samples. Consequently, no single existing technology can simultaneously satisfy all requirements, including high-precision quantification, non-destructiveness, low cost, strong anti-interference capability, and universal adaptability to complex real-world food matrices. Multi-technology fusion, which combines the complementary merits of different sensing modalities, therefore represents a promising solution.

Over the past decade, rapid detection technologies for food freshness, including sensory simulation, biosensing, and spectroscopic imaging, together with their deep integration with artificial intelligence, have achieved substantial progress. The coupling of diverse sensing platforms with AI models has markedly improved detection sensitivity, speed, and automation, thereby reshaping food safety and quality assessment. However, the transition from controlled laboratory environments to complex real-world settings still faces a series of severe challenges, which collectively constitute the principal bottlenecks to industrialization and large-scale application [109,110]. These limitations can be attributed to three interrelated factors. First, the robustness of sensory-simulation technologies varies with environmental conditions, including interference from temperature, humidity, and complex background volatile organic compounds [111,112]. Second, biosensors remain dependent on recognition elements (e.g., enzymes, antibodies, and aptamers) whose performance is limited by the susceptibility of natural enzymes to inactivation, the high cost and batch-to-batch variability of antibodies, and the complex validation procedures required for aptamers [113]. Third, the adoption of spectroscopic imaging is restricted by the high cost of hyperspectral sensors and the absence of common operational standards, which further complicates data acquisition, feature extraction, and data storage [114]. More broadly, a common translational gap persists across all platforms: the trade-off between analytical rigor and practical operability remains unresolved. While AI and multi-modal fusion have enhanced predictive performance, they have simultaneously introduced new challenges in model generalizability, data standardization, and system complexity.

Looking ahead, rapid detection technologies for food freshness will evolve toward greater integration and intelligence, and future research should prioritize the following directions. (1) AI-driven next-generation sensing systems, through the tight integration of dynamic sensor-array optimization strategies with intelligent algorithms [115]. It should be noted that, although AI has opened novel avenues for food freshness detection and delivered promising gains in both analytical accuracy and operational efficiency, several aspects warrant continued scrutiny: model performance is intrinsically linked to the representativeness of training data and may degrade when models are deployed across divergent scenarios; the explainability of deep-learning decision processes remains to be refined; discrepancies in data-acquisition conditions across instruments typically necessitate targeted adaptation to ensure reliable cross-instrument model transfer; and standardized certification frameworks for AI-assisted detection are still under development. These issues reflect the inherent trajectory of emerging technologies as they transition from laboratory validation to large-scale deployment and are being systematically addressed through ongoing research. (2) Stimuli-responsive smart polymers for smart packaging, which integrate freshness monitoring with active preservation functions (e.g., antibacterial and antioxidant activity). (3) Biomimetic nanozymes with higher stability, selectivity, and catalytic activity replace or supplement natural enzymes, thereby reducing sensor cost and improving environmental tolerance [116]. (4) Enhanced standardization and collaboration, i.e., establishing unified standards for rapid detection technologies across diverse food samples and strengthening cross-sector collaboration, so as to fully unlock their application potential in the comprehensive management and control of food quality and safety [117,118].

**Table 5:** Summarized comparison of different technologies on advances and limitations.

Category	Technology	Analytical Principle	Target Analytes	Response Time	Analytical Performance	Advantages	Limitations	Portability	Technology Maturity	Commercialization Status	Scalability	Economic Feasibility
Sensory Simulation	Electronic Nose	Gas sensor array captures volatile organic compounds (VOCs), converts them into electrical signals, and combines them with pattern recognition algorithms	VOCs, TVB-N, freshness grade, shelf life	Seconds to minutes	Classification accuracy 96–99.7%; TVB-N prediction R^2^ = 0.92–0.94	Non-destructive, rapid, olfactory simulation, suitable for dynamic prediction	Sensor drift, complex calibration, matrix interference, lack of unified standards	Medium-High (portable systems available)	Medium-High	Commercial products exist; most applications are still in lab validation and pilot stage, lacking unified industry standards	Medium-High.	Medium.
	Electronic Tongue	Biomimetic taste receptors convert chemical information from food extracts into electrical signals with pattern recognition	Microbial quality, freshness, flavor compounds	Relatively fast (minutes)	Fish storage day recognition rate: 97.22%	Objective, alternative to sensory evaluation, multi-indicator correlation	Sensor drift, calibration shift, poor reproducibility, insufficient standards	Medium	Medium	Commercial products available, freshness detection applications limited, mostly research use	Medium. Bulky equipment.	Medium-Low.
	Machine Vision	Captures and quantifies external color/texture/morphology changes, combined with deep learning for automatic feature extraction	Color grade, freshness, pH, TVB-N, volatile amines	Rapid (30 s to sub-second)	TVB-N prediction R^2^ = 0.91–0.94; shrimp/fish freshness classification 99.25%	Intuitive, non-destructive, efficient, smartphone-compatible platforms	Susceptible to illumination, surface phenotype only, individual variation interference	High (smartphone platforms)	High	Highly commercialized, fresh produce recognition apps growing fast	High. Integrable.	High. Low hardware cost
		Biorecognition elements capture spoilage signals and convert them into measurable optical signals (color, fluorescence intensity, refractive index)	Cadaverine, pH, spoilage gases, biogenic amines	Rapid (minutes)	High sensitivity, fast response	Real-time, rapid, non-destructive, intuitive signals	Photobleaching, background fluorescence interference, limited quantitative capability	Medium-High	Medium	Freshness detection mostly in R&D and pilot stage; smart packaging indicators have limited commercialization	Medium.	Medium.
		Biomolecular changes converted to surface mass variations, detected via resonance frequency shift in piezoelectric crystals	Ethylene, TMA	Real-time	High-sensitivity mass detection	High sensitivity, real-time response, miniaturization potential	Insufficient selective and durable materials, complex signal processing	Medium	Low-Medium	Food freshness detection is essentially in the lab research stage, with no mature commercial products	Low-Medium.	Low.
Biosensing	Electrochemical Biosensors	Biorecognition elements immobilized on electrode surface interact with targets to produce current/potential changes	XA, HX, biogenic amines	Rapid (~1 min)	High selectivity, long-term stability, good recovery rates	Fast, sensitive, quantitative, relatively low cost	Electrode fouling, sample pretreatment needed, complex matrix interference	Medium	Medium	food freshness sector has startup products and test strips, some in pilot and small-scale commercial use	Medium-High.	Medium-High.
	Enzyme-based Biosensors	Enzymes (natural/nanozymes) as recognition elements catalyze target reactions to produce measurable signals	HX, XA, histamine, TVB-N	Rapid	High specificity, integrable with paper microfluidics	Simple and fast, high specificity, multifunctional integration	Poor natural enzyme stability, high cost, nanozyme selectivity needs improvement	Medium-High (paper-based/flexible electrodes)	Medium	Food freshness (HX, histamine) detection mostly in R&D/pilot; paper-based microfluidic products emerging	Medium-High.	Medium.
	Antibody-based Biosensors	Antigen–antibody specific binding recognizes spoilage markers, converted to color/fluorescence signals	Histamine, spoilage microorganisms	Rapid	High selectivity and sensitivity (comparable to fluorescent labeling)	High selectivity, sensitive, mature immunoassay strategies	Limited antibody stability, high cost, potential cross-reactivity	Medium (microarray chips)	Low-Medium	Real-time food freshness detection is limited, mostly lab methods	Medium.	Medium-Low. High antibody cost.
	Nucleic Acid Aptasensors	Aptamers (in vitro selected oligonucleotides) specifically bind target ligands with signal amplification strategies	ATP, biogenic amines	Rapid	High specificity, dual-mode detection, reliable assessment	Stable and modifiable, low cost, high designability	Complex screening process, signal amplification needed, insufficient standardization	Medium	Low	Mainly in lab research and proof-of-concept stage, few startups exploring commercialization	Low.	Low. High R&D cost.
	Microbial-based Sensors	Detects microbial activity or metabolites (ammonia, amines, ATP) to indirectly reflect freshness	Ammonia, amines, ATP, microbial metabolites	Moderate (minutes)	Indirect monitoring, strong correlation with spoilage process	In situ, reflects real microbial status, integrable with smart packaging	Limited specificity, slower response, microbial cultivation requirements	Medium (smart packaging films)	Low	Sensors directly detecting microbial activity or metabolites, mostly in R&D	Low-Medium.	Low-Medium.
Spectroscopy & Imaging	Near-Infrared Spectroscopy (NIR)	Absorption and scattering of NIR radiation by hydrogen-containing functional groups (water, fat, protein)	TVB-N, pH, biogenic amines, moisture, fat	Rapid (seconds)	TVB-N prediction R^2^ = 0.92–0.9342; pH prediction R^2^ = 0.9253	Non-destructive, rapid, high maturity, fiber-optic deployable, portable	Requires large calibration sets, severe spectral overlap, moisture-sensitive	High (portable spectrometers)	High	Highly commercialized, portable devices fast growing	High.	High.
	Hyperspectral Imaging (HSI)	Simultaneously acquires spatial morphological information and continuous spectral data, integrating imaging and spectroscopy	Moisture, pH, color, TVB-N, TVC	Moderate (slow data processing)	Vegetable classification >80%; chicken breast TVB-N/TVC Rp^2^ = 0.904/0.9499	Spectrum-image integration, rich information, spatial distribution analysis, mechanistic interpretability	Large data volume, expensive equipment, complex calibration, limited real-time performance	Low-Medium	Medium	Partially commercialized, mainly for research and high-end quality inspection	Medium.	Low-Medium. System cost is high.
	Raman Spectroscopy	‘Fingerprint’ scattering signals from laser-molecule interaction, monitoring chemical bond changes during spoilage	TVB-N, TBARS, proteins, purines, lipid oxidation	Very fast (0.44 s)	Sea bass fillet freshness classification 90.6%	Molecular-level specificity, non-destructive, fast, minimal water interference	Strong fluorescence background interference, weak signals, poor reproducibility, SERS substrate uniformity issues	Medium-High (portable Raman)	Medium	Food freshness detection mostly research/pilot; SERS substrates are a key bottleneck	Medium.	Medium.
	Fluorescence Spectroscopy	Specific wavelength excitation of endogenous fluorophores emits fluorescence for indirect freshness assessment	TVB-N, pH, biogenic amines (phenethylamine, cadaverine)	Very fast (sub-second)	Grass carp fillet freshness detection > 95%; high-precision tilapia TVB-N prediction	Fast, sensitive, device miniaturization, UV-LED compatible	Indirect measurement, photobleaching, matrix interference, limited absolute quantification	High (portable UV-LED systems)	Medium	Partially commercialized, UV-LED portable fluorescence systems available, food freshness detection mostly in R&D	Medium-High.	Medium-High.

## Data Availability

No new data were created or analyzed in this study. Data sharing is not applicable to this article.
